# Eyes Toward the Clinic: Selective Inhibition and Degradation Approaches to Bromodomain‐Containing Proteins

**DOI:** 10.1002/cbic.70443

**Published:** 2026-06-30

**Authors:** Cole R. Scholtz, William C. K. Pomerantz

**Affiliations:** ^1^ Department of Chemistry University of Minnesota – Twin Cities Minneapolis Minnesota USA

**Keywords:** bromodomain, clinical trials, epigenetics, PROTAC

## Abstract

Bromodomain‐containing proteins serve as epigenetic regulators, where bromodomains recognize acetylated proteins and histones to drive changes in gene expression. Due to their regulatory role in gene expression, bromodomain‐containing proteins have served as important targets for drug‐discovery efforts, in particular the bromodomain and extra‐terminal (BET) family proteins. The selective targeting of these proteins through both inhibition and targeted protein degradation has revealed diverse biological mechanisms. For BET bromodomain‐containing proteins, we discuss the unique biological effects of selective inhibition and degradation for efficacy and clinical safety concerns for both BET inhibitors that have entered Phase III clinical trials and recent BET degrader clinical candidates. We also describe the current inhibitory and targeted protein degradation approaches of non‐BET bromodomain‐containing proteins. This review summarizes current approaches of bromodomain‐targeting inhibition and degradation to provide insight on molecular mechanisms and updates the clinical progress of these modalities as the field awaits the approval of the first bromodomain‐targeted therapeutic.

## Graphical abstract

The function of bromodomain‐containing proteins in regulating gene transcription is reviewed here, including the development of chemical probes and targeted protein degradation modalities (e.g., PROTACs). This extends to clinical efforts targeting the BET bromodomain‐containing proteins. Challenges of selectivity, future prospects, and the differential biological effects between bromodomain inhibition and protein degradation are discussed.

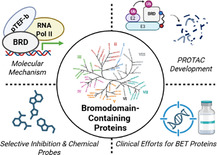

## 1 Introduction

While all somatic cells contain the same DNA sequence, the regulation of gene expression independent of DNA sequence plays an important role in determining cellular identity, proliferation, and response to external stimuli [1]. The field of epigenetics focuses on heritable changes in gene expression or phenotypic changes independent of gene sequence [2, 3]. Epigenetic mechanisms govern gene expression through a diverse set of pathways and the alteration of DNA organization for regulating transcription. Examples of epigenetic mechanisms include (i) the modification of DNA bases [4], (ii) the regulation of non‐coding RNA [5], and (iii) the post‐translational modifications to histones [6, 7]. These epigenetic mechanisms provide control within the cell to regulate both transcriptional activation and gene silencing, where the mechanisms involved vary between tissue type and are vital to cellular differentiation [8]. Due to their important role in controlling gene transcription, the proteins involved in regulating these epigenetic pathways are of growing interest in drug discovery, as dysregulation of these dynamic pathways can lead to diverse disease states such as cancer, inflammation, fibrosis, neurodegenerative, and infectious diseases [9, 10, 11].

**FIGURE 1:**
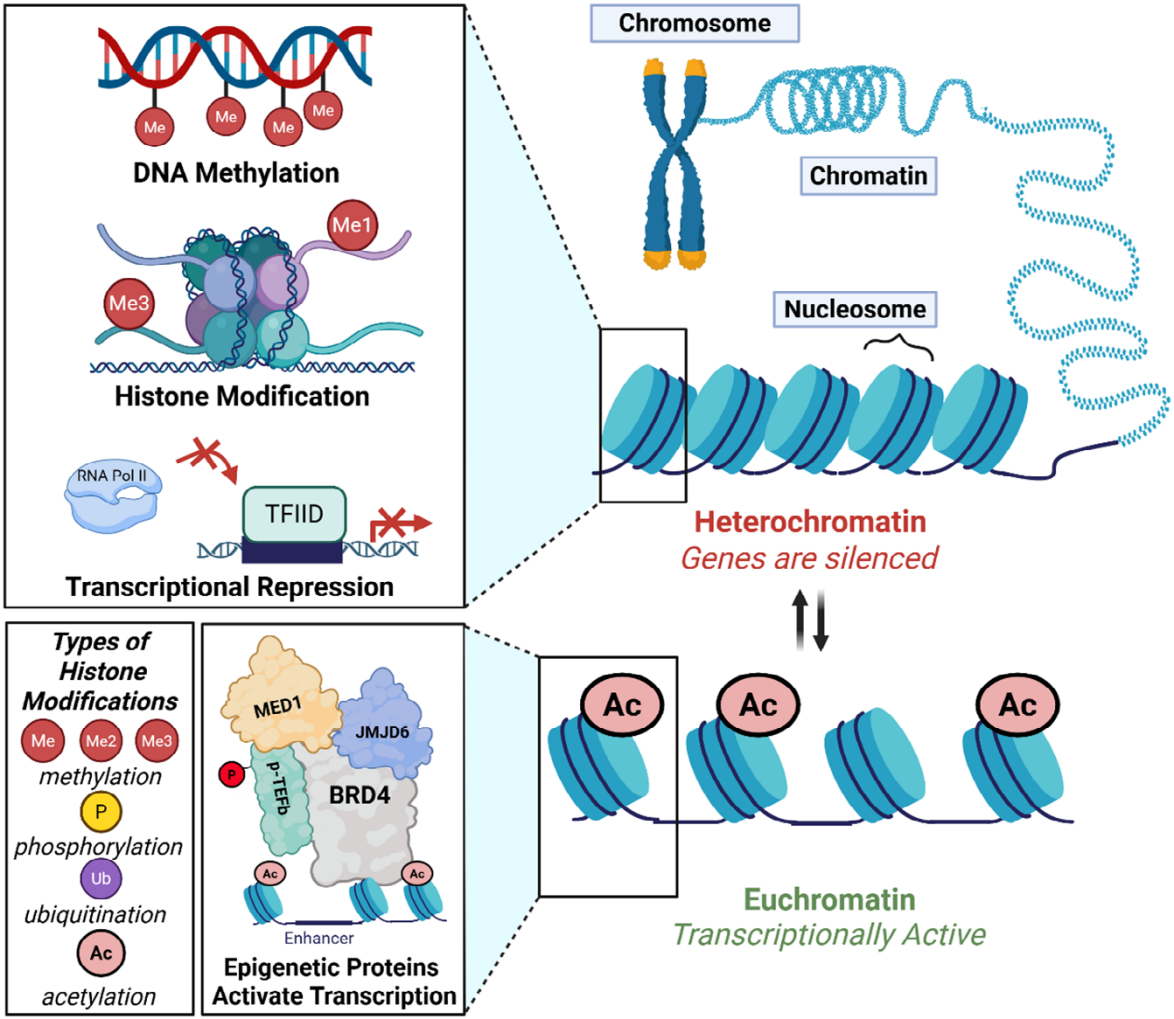
Epigenetic mechanisms regulate gene expression through post‐translational modification of DNA and histones (top). Epigenetic modifications and mechanisms present at the DNA and histone modification level (bottom) and the recruitment of transcriptions factors (e.g., BRD4) serve to recruit additional transcriptional machinery and control gene regulation. In doing so, these mechanisms control DNA transcription through a variety of mechanisms including engaging transcription factors and their interactions with nucleosomes.

Catalyzed by DNA methyltransferases, DNA methylation involves the addition of methyl groups directly to DNA nitrogenous bases. One example is the methylation of cytosine‐guanine (CpG) islands, which represents a stable form of gene silencing [12]. CpG islands are found at 70% of gene promotor regions, where methylated nucleotides serve as a repressive mark to reduce the interactions between DNA and activating transcription factors, also serving to recruit transcriptional repressors (Figure 1) [13, 14, 15]. Additionally, hypermethylation of tumor suppressor genes and hypomethylation of proto‐oncogenes contribute to tumor growth [16]. As such, DNA methyltransferases were explored as clinical targets in combination with traditional therapies in solid tumors to enhance radiation sensitivity with high fidelity [17]. A second method of epigenetic regulation is through long non‐coding RNA (lnc‐RNA). Though RNA‐regulated epigenetic regulation is still being fully investigated, lnc‐RNA can serve as a guide to recruit or repress transcription factors, and serve as scaffolding for larger chromatin structures [18]. Further, complexes that target non‐protein‐encoding nascent transcripts can mediate stable changes in gene expression, whereas shorter ncRNAs maintain a persistent memory of silenced genes based on self‐reinforcing feedback loops [19].

The structure and covalent modification of histone proteins also represent a significant and dynamic portion of epigenetic regulation. Nucleosomes are comprised of a histone octamer core with 147 base pairs of DNA (∼1.7 turns) around each histone octamer [20]. Each octamer contains two protein copies of H2A, H2B, H3, and H4 histone proteins. In the case of histone proteins, unstructured N‐terminal residues and termini protrude from the histone surface, providing sites for post‐translational modification [6, 21]. Common types of post‐translational modifications include methylation, acetylation, phosphorylation, and ubiquitinylation on a variety of residues [22, 23], though additional modifications have been identified and are continuing to be discovered (Figure 1) [24, 25, 26]. Post‐translational modifications of histones act as epigenetic marks, which recognize transcriptional regulators and chromatin remodeling factors to regulate downstream transcription and modulate heterochromatin (inactive) and euchromatin (active) states. For example, acetylation of H3K9Ac and H3K27Ac correlates with transcriptional activation and works in part through reducing electrostatic interactions with nucleosomal DNA and reader domain recruitment [24, 27, 28]. While histone‐DNA interactions are part of transcriptional activation, transcription factors with reader domains (e.g., bromodomains or PHD domains) also serve an important role leading to transcriptional initiation. Transcriptional activation and elongation through these transcription factors is performed through a variety of bromodomain‐containing proteins, such as BRD4 [29, 30], BRD2 [31], CBP/p300 [32, 33], or through chromatin remodeling with SWI/SNF [34]. Histone methylation is more complex, as it can be both repressive and activation dependent on the methylation pattern, as seen for H3K27Me_3_ and H3K4Me_3_, respectively.

While numerous histone modifications have been identified, acetylation is one of the best characterized and most abundant posttranslational modifications [35]. Importantly, histone acetylation is a dynamic and reversible process, where the inhibition of proteins essential in the recognition of epigenetic marks are important therapeutic targets. Subsets of these proteins contain bromodomains, which recognize acetylated histones and subsequently recruit auxiliary transcriptional machinery to remodel epigenetic landscapes [36, 37]. In cancer, the dysregulation of bromodomain‐containing proteins can lead to activation of oncogenic proteins [38, 39, 40]. Bromodomains are also found in necessary chromatin remodeling complexes, leading to their pivotal role in governing transcription [41, 42]. As such, proteins involved in histone acetylation (such as bromodomain‐containing proteins, histone deacetylases, and histone acetyl transferases) are particularly attractive for drug discovery efforts. In addition to drug discovery, chemical methods to study these processes through chemical probe development or chemical biology innovations help to define the field of chemical epigenetics. YEATSs domains are a second domain which recognize histone acylation, and are additional targets for drug discovery, but are outside the scope of this review and have been reviewed elsewhere [43].

## 2 Structural Analysis of Bromodomain‐Containing Proteins

**FIGURE 2:**
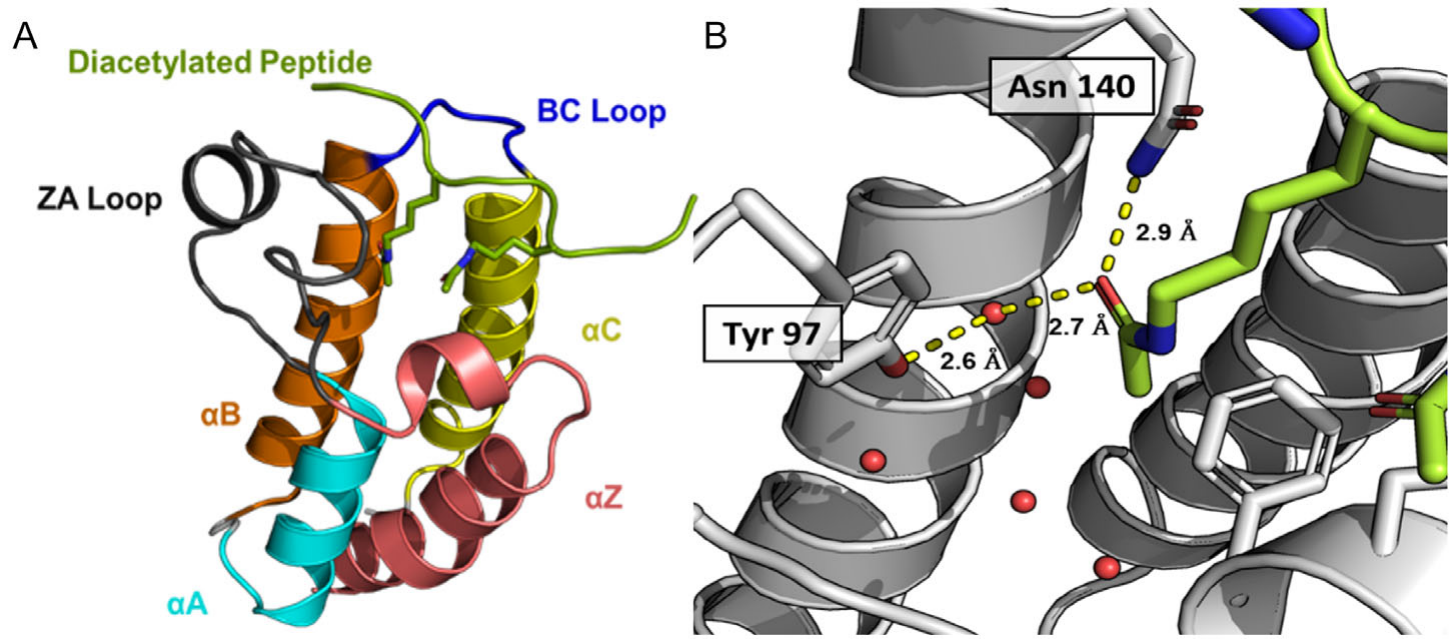
Co‐crystal Structure of BRD4(D1) with H4K5AcK8Ac peptide, (A) labeled structural components of BRD4(D1) bound to diacetylated H4 peptide (PDB: 3UVW). (B) Diacetylated H4K5Ac8Ac peptide (lime) interacting with binding site of representative bromodomain, BRD4(D1) (gray). Hydrogen bonds shown as dashed yellow lines in Angstroms (Å). Waters shown as red spheres. (PDB: 3UVW).

Bromodomains are specific domains that recognize N‐ε‐acetylated lysine (Kac), where they bind to acetylated histones and acetylated proteins, including transcription factors to promote active gene transcription [28, 44]. Due to their significant role in governing downstream gene expression, inhibition of the interaction between bromodomains and acetylated lysine has been identified as a potential therapeutic target in a variety of cancers, inflammation, and autoimmune disorders [38, 45, 46]. The bromodomain motif is an evolutionarily conserved substructure consisting of ∼110 amino acids [47]. Bromodomain structure is comprised of left‐handed four‐helix bundles (αZ, αA, αB, and αC) linked by two loop regions (ZA and BC loops, Figure 2A) [48]. The four helices along with the two loop regions form a deep binding pocket suitable for Kac recognition [47]. Bromodomains have high sequence homology, with 61 bromodomains across 46 unique human proteins, and are classified into eight sub‐categories [47, 49]. Bromodomains canonically recognize acetylated marks through a water‐mediated hydrogen bond with tyrosine and a direct hydrogen‐bond with an asparagine residue. Despite the structural similarity between the bromodomain motif, a small fraction of bromodomains present a different residue other than asparagine (e.g., a threonine or tyrosine residue) to aid in acetyl lysine recognition (Figure 2B). These non‐canonical bromodomains also exhibit unusual ZA‐loop architecture, as seen in PHIP(2), BRWD(3), WDR9(2), MLL, ASH1L, TRIM28 [49, 50, 51]. Despite these structural differences, these non‐canonical bromodomains still bind histones in an acetylation‐dependent manner, suggesting alternative mechanisms to natural ligand specificity. Notably, MLL recognizes very few acetylated histone peptides, but does recognize acetylated H2A and H2B [45, 52]. Bromodomains generally bind to acetylated histone peptides with affinities in the mid‐to‐low micromolar ranges, though proteins with multiple bromodomains can recognize acetylated histones with nanomolar affinities [45].

A structured water network between four and five water molecules exists within the hydrophobic core of the bromodomains [47, 53]. In Class I and Class II bromodomains, these structured waters are encapsulated by a hydrophobic WPF shelf, which acts as a set of gate‐keeper residues. This structural motif is also present in the Class VII bromodomain TAF1, though none of the other Class VII bromodomains [47]. Similarly, a subset of Class III bromodomains (such as PHIP(D2), CBP, and p300) show a similar hydrophobic set of gatekeeper residues, though not BRD8. Structural analysis of human bromodomains focusing on the conserved set of structured water molecules has focused on the druggability of bromodomains [54]. Where most native and synthetic ligand interactions do not replace these structured waters, selectivity can be gained for ligands that perturb or reorganize these waters [42, 55, 56, 57, 58]. In several cases, in silico analysis of the energetics relating to the structured waters has revealed that higher energy waters can be displaced, leading to preferential selectivity over other bromodomains [59]. Displacement of these structured waters has been observed in bromodomains of ATAD2 [58], SMARCA2/4 [60, 61], BRD7/9 [55, 62], and the N‐terminal bromodomain (D1) of BRD4 [57]. Work from our lab has capitalized on the structured water displacement, leading to the development of BRD4‐D1 selective inhibitors over the other members of the BET bromodomains [56, 63]. The development and study of these structured waters are aided by the ample structural data available, where a high‐resolution X‐ray or NMR structure is available for 77% of bromodomains [47]. Within these structures, the bromodomain and extra terminal (BET) family accounts for ∼40% for all structures in the Protein Data Bank (PDB). Indeed, there is a distinct bias in the structural characterization of bromodomains, as Classes I, II, IV, VI, and VIII have been completely or nearly completely characterized, which is in contrast to Classes III, V, and VII, which lack structures for several family members and represent a future need for structural analysis [64].

## 3 Biological Functions of Bromodomain‐Containing Proteins

**FIGURE 3:**
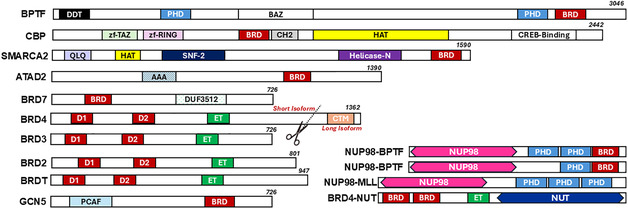
Domain organization of a subset of bromodomain containing‐proteins and model fusion proteins. Domain organization is representative bromodomain‐containing proteins. Though individual proteins can contain multiple bromodomains (red), additional functional domains enable complex transcriptional machinery. Lengths are shown on the right in amino acids. BRD or D1/D2 (red) = bromodomains, ET (green) =extra‐terminal domains. CTM (peach) = C‐terminal motif. HAT (yellow) = histone acetyl transferase. NUP98 (pink) = Nucleoporin 98. NUT (blue) = nuclear protein in testis. DDT (black) = DNA‐binding homeobox‐ containing proteins and the Different Transcription. PHD (cyan) = Plant homeodomain. BAZ (white) = bromodomain adjacent to PHD zinc finger. zf‐TAZ (light green) = Transcription adaptor putative zinc finger. zf‐RING (light pink) = Really interesting new gene, serves as structural motif and allosteric activator for ubiquitination. CH2 (gray) = cysteine/histidine‐rich region 2. QLQ (light purple) = conserved Gln‐Leu‐Gln motif. PCAF (light blue) = 300/CBP‐associated factor.

While the structure and sequence of bromodomains themselves are highly conserved, the structures and functions of bromodomain‐containing proteins are diverse, as are the domains surrounding them. Several proteins contain multiple bromodomains, which may increase the overall binding affinity for poly‐acetylated marks [47]. Due to their complexity, bromodomains are organized into eight classes, where bromodomains in Class I, II, III, and IV function to control lysine acetylation, lysine methylation, chromatin remodeling, and chromatin assembly, respectively. Likewise, bromodomains in Classes V, VI, VII, and VIII regulate transcriptional activation, protein ubiquitination, nuclear transcriptional activation, and control of cell shape, respectively [65]. While the BET proteins contain tandem bromodomains, the N‐terminal domain of Brdt (Brdt‐D1) has simultaneous and cooperative binding to the diacetylated mark H4K5acK8ac, suggesting similar interactions for other members of the BET family through a variety of biophysical and crystallographic techniques [66]. In the same work, Morinière et al. also suggest similar interactions for CBP/p300 and TAF1, a trend that is currently being explored among bromodomain‐containing proteins [67, 68]. Bromodomain subunits are often surrounded by additional domains to modulate downstream transcription through the recognition of additional epigenetic marks or through the recruitment of additional transcriptional machinery [69]. In the example of BRD4, the presence of the C‐terminal motif (CTM) regulates the downstream transcription through direct interactions with P‐TEFb (Figure 3) [70, 71]. The absence of this motif, present in the BRD4‐S isoform, causes distinct biological effects in breast cancer models, particularly in its interactions with the *CST4* promoter [72]. The largest subunit of the NURF chromatin remodeling complex, BPTF, contains both a bromodomain as well as a PHD domain [73]. Due to these tandem binding domains, BPTF binds both H4K16Ac and H3K4Me_3_ within the same nucleosome unit, where BPTF's bidentate binding to a singular nucleosome dictates nucleosome selectivity [74, 75]. Recent developments from Platt et al. take advantage of neighboring PHD domains and bromodomains of TRIM24 to develop peptide‐drug conjugates with high efficacy [76]. Bivalent small‐molecule inhibitors have also found success targeting neighboring domains within the BET family, where the bivalent inhibitor MT1 was able to engage the BET bromodomains simultaneously and resulted in increased cellular efficacy [77]. Indeed, the interplay between epigenetic regulatory domains and dynamic protein–protein interactions provides several possible mechanisms for regulating transcription through the recognition of epigenetic signaling.

Consistent with their role in regulating gene expression, bromodomain subunits are often found with histone acetyl transferase (HAT) complexes and chromatin remodeling complexes (Figure 3) [78]. The mammalian proteins CREB binding protein (CBP) and E1MSA‐associated protein p300 (p300), both members of Class III, each have a bromodomain and a HAT domain in addition to modulating protein–protein interactions. CBP and p300 represent homologous proteins that are similar in their structural and functional similarity [79]. These proteins play an important role in disease, as both are enriched at enhancer and promoter regions of the genome [80]. As such, both CBP and p300 proteins have been implicated in prostate cancer and acute myeloid leukemia [79, 80, 81]. Despite the similarity in these proteins, p300 loss prevents retinoic acid‐induced differentiation, whereas CBP loss does not [82]. Further, heterozygous knockout of CBP in mice resulted in hematopoietic failure and hematologic malignancies, whereas heterozygous knockout of p300 showed no discernible change in phenotype, suggesting distinct roles in their biological function [83]. This finding is consistent with reports by Durbin et al. in neuroblastoma, where small‐molecule‐induced p300 degradation modulates the architecture of enhancer regions for the regulation of the core regulatory circuit of transcription factors [84]. This trend is also seen in other oncogenic fusion proteins, where the NUP98‐BPTF fusion proteins retain the C‐terminal bromodomain with either one or two copies of the neighboring PHD domain being retained [85, 86]. NUP98 also forms a fusion protein with MLL, which retains the majority of the MLL reader domains and protein structure. This is despite BPTF and MLL belonging to different classes of bromodomains (Class I and VI, respectively). Although these fusions are rare, the role of NUP98 fusion proteins in dysregulating the *HOXA* gene clusters is associated with acute leukemia and other malignancies [87]. Other examples of oncogenic fusion proteins include the BRD4‐NUT fusion protein, where this fusion protein alone drives an aggressive squamous carcinoma with limited therapeutic options (Figure 3) [88]. While bromodomains serve to both anchor proteins to chromatin and recognize acetylation patterns [78, 89], their complex role in managing gene expression through protein–protein interactions warrants further investigation into their role in regulating disease for validating as therapeutic targets.

Aside from recognition of acetylated histones, the BET family of bromodomains (Class II) was demonstrated to recognize additional acetylated motifs in non‐histone proteins, including transcription factors such as GATA1 [90], TWIST [91], and the p65‐subunit of the nuclear factor‐κB (NF‐κB) [92, 93]. In doing so, the BET family of proteins affects the transcription of downstream target genes through stabilizing the interaction between the transcription factor and acetylated chromatin. The transcription factor GATA1 is an megakaryocyte‐specific transcription factor, where two conserved lysine residues (K312 and K315) are acetylated. These acetylated lysine residues are recognized by BRD3, an interaction which is essential for mature red blood cell production [94]. A similar interaction has been characterized between BRD4 and TWIST, where the acetylation of two conserved lysine residues on TWIST (K73 and K76) led to BRD4 recognition of TWIST in cells [91]. The interaction between BRD4 and TWIST is important for controlling tumor invasion and tumorigenicity in breast cancer cells. The p65‐subunit of NF‐κB is acetylated (K310) by the HAT domain of CBP/p300 [95, 96]. Moreover, all three proteins, NF‐κB, CBP/p300, and BRD4, can form a complex at super enhancers, driving inflammation, providing additional levels of control and therapeutic targets [97, 98].

## 4 Inhibitor Development for Non‐BET Bromodomain‐Containing Proteins

### 4.1 Emerging Inhibitor Development for Class V, VI, and VII, and VIII Bromodomains

**FIGURE 4:**
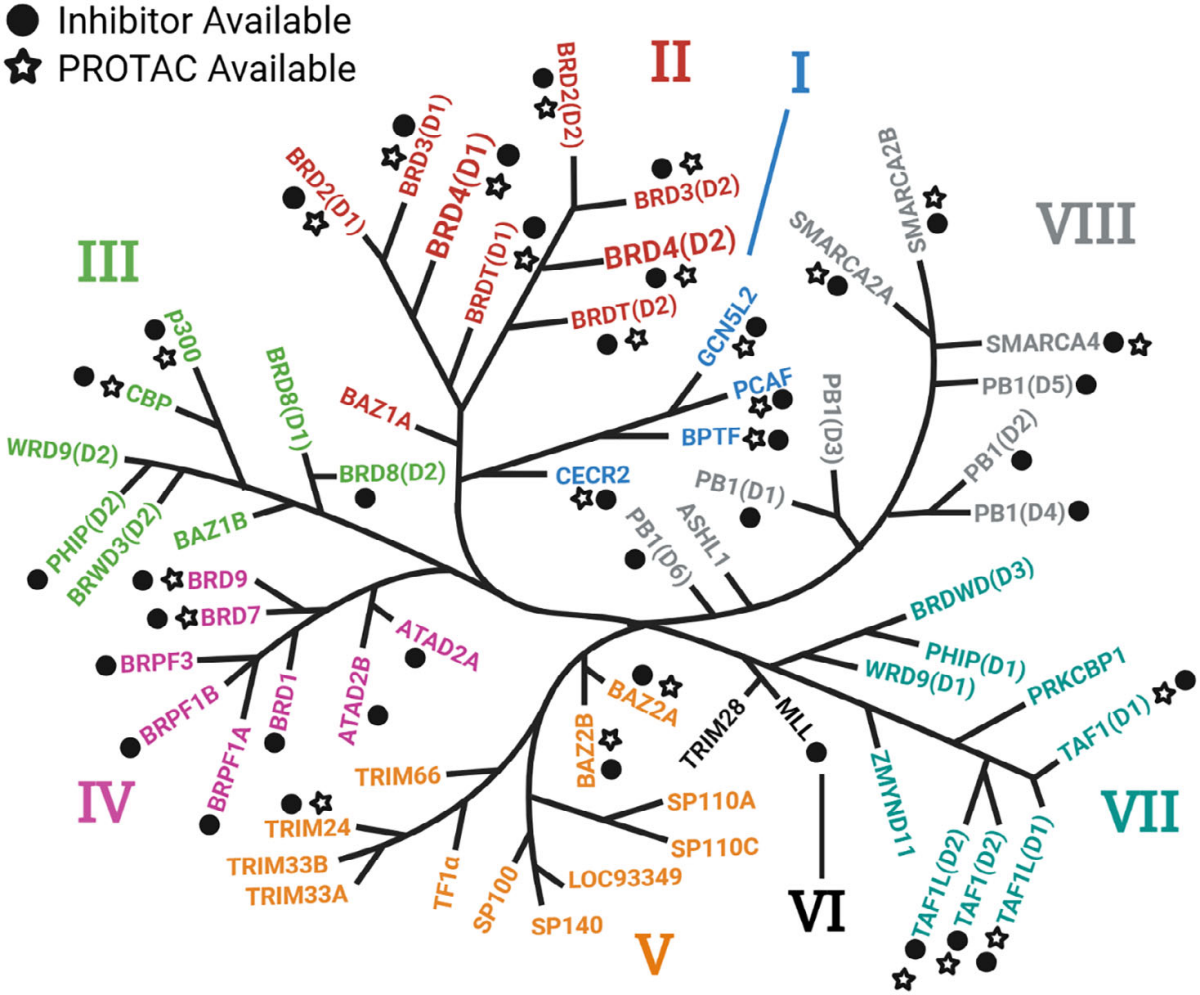
Phylogenetic tree of bromodomains by structural similarity. Phylogenetic tree originally reported by Filippakopoulos et al., where bromodomains classes are given by roman numerals (I–VIII) [43] Small‐molecule bromodomain inhibitors available marked with a black circle, targeted protein degraders (PROTACs) are noted with a white star.

**FIGURE 5:**
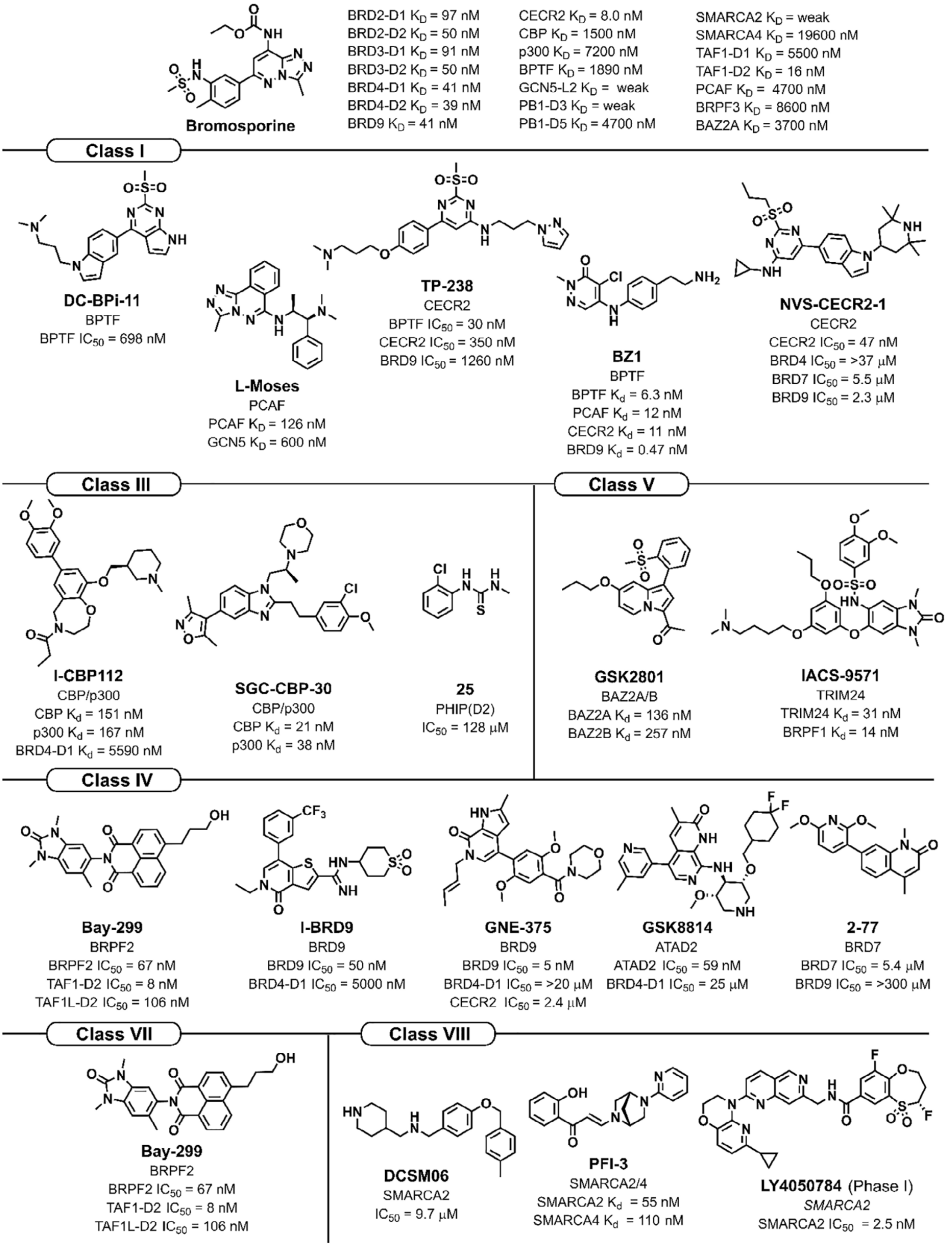
Structure of representative inhibitors targeting non‐BET bromodomains. Reported affinities of inhibitors to their respective bromodomains are also shown. Affinities reported range from a variety of assay formats, and caution should be used when directly comparing absolute affinities across multiple assay formats.

Although numerous bromodomain‐containing proteins have been targeted for inhibitor development, a significant portion of bromodomains remain without potent inhibitors [99]. The families that remain largely undeveloped from an inhibitor development lens include the Class V bromodomains, Class VI bromodomains, and Class VII bromodomains (Figure 4). Largely, these classes of bromodomain‐containing proteins have fewer than three inhibitors available that target their bromodomains. While bromosporine (Figure 5) serves as a weak‐to‐moderate potency inhibitor for many of these bromodomains, more specific inhibitors are needed in this area to readily investigate biological mechanisms in disease [100]. The reduced number of inhibitors for these bromodomains is likely a result of them being less structured and flexible in nature (e.g., TRIM28, Class VI).

Alternative explanations to the lack of availible inhibitors include their function in large complexes, where bromodomain inhibition may be insufficient to drive biological effects (as seen in SMARCA2 with BAF complexes, Class VIII) [101]. However, it is worth noting that recent bromodomain‐targeting molecules have been identified for a few of these bromodomains, specifically through high‐throughput screening [60, 102]. Inhibitors developed toward these bromodomains include inhibitors from Genentech, targeting the second domain of TAF1 and leading to the development of GNE‐371 [103]. Further, pan‐bromodomain inhibition of Class VIII bromodomains of PB1(1–6) and SMARCA2/4 have been useful in elucidating their function in adipogenesis, where inhibition of these bromodomains led to the downregulation of adipogenesis biomarkers and impaired the differentiation into adipocytes [104]. Selective inhibitors targeting PB1's second and fifth bromodomains, PB1(2) and PB1(5), have also been reported in the form of LM146, which was >30‐fold selective for PB1(2) and PB1(5) over the bromodomains of SMARCA2 [105]. It remains to be seen whether the selective inhibition of PB1 will elucidate additional clarity of its biological functions.

### 4.2 Potent Bromodomain Inhibitors in Class I, II, III, and IV

Of the remaining classes of bromodomain‐containing proteins (Class I, II, III, and IV), multiple potent inhibitors have been developed toward the CBP/p300 bromodomains. The first potent inhibitors for CBP/p300 were developed by Hay et al. based on a 3,5‐dimethylisoxazole (SGC‐CBP‐30), leading to a reduction in CBP‐mediated p53 activity in a colon cancer model [106]. At the same time, dihydroquinoxalinone‐based inhibitors were also developed. However, SGC‐CBP‐30 retains weak inhibition of BRD4, masking the true biological function. As such, SGC‐CBP‐30 motivated the development of more selective ligands targeting CBP/p300, which would reveal indirect CBP/p300‐mediated effects on c‐Myc [107]. Inhibition of the CBP/p300 bromodomains through I‐CBP112 also resulted in cellular reprogramming through chromatin accessibility (Figure 5) [81]. However, inhibitor development for other Class III bromodomain‐containing proteins remains mostly behind that of CBP/p300, as only low‐potency ligands (discovered through fragment screens) have been reported for bromodomains such as PHIP(2) [108]. One notable exception to this is targeting BRD8, where nanomolar ligands that are ∼30‐fold selective for BRD8(2) over BRD8(1) have been described [109].

Discovery efforts targeting Class I bromodomain‐containing proteins has produced potent small molecule inhibitors, leading to the development of CECR2 [110], PCAF/GCN5 [111], and BPTF [73, 112]. Class I inhibitors have since reached nanomolar potencies, including NVS‐BPTF‐1 (*K*_D_ = 70 nM) and BI‐7190 (*K*_D_ = 3.5 nM) [113, 114]. These inhibitors have been vital for deciphering the role of non‐BET bromodomains in DNA damage response, HIV replication, and neuroblastoma [115], respectively [116, 117, 118, 119, 120]. Investigation into inhibitor development focusing on the Class IV bromodomains has also yielded highly potent inhibitors, specifically in the case of BRD7 and BRD9. The structural similarity of BRD7 and BRD9 represents a significant challenge for designing selective inhibitors (similar to challenges faced with BET bromodomains). However, selective inhibitors for both BRD7 and BRD9 have been reported. For BRD7, the potent and cell‐active compound 2–77 was used to validate BRD7's function in both the PBAF complex and androgen‐positive prostate cancer, where BRD7 bromodomain inhibition impairs the function of the PBAF complex [121]. The first selective inhibitor for BRD9 was developed by GSK (I‐BRD9) through exploiting differential hydrogen‐bonding patterns, which resulted in the downregulation of genes related to cancer and immune pathways [42]. The development of other BRD9‐targeting inhibitors soon followed, where both Genentech/Constellation Pharmaceuticals and Boehringer Ingelheim developed GNE‐375 and BI‐7273, respectively [55, 122]. The latter displayed moderate anti‐tumor activity in acute myeloid leukemia (AML) xenograft models, though the development of BRD9‐selective PROTACs increased efficacy by 1000‐fold, as discussed in Section 9 [123]. These stark differences between therapeutic modalities helped to elucidate the role of BRD9's bromodomain in the SWI/SNF complex, where bromodomain inhibition may be ineffective in these large transcriptional complexes.

Additionally, nanomolar ligands for the Class IV bromodomains ATAD2 and BRPF have also been developed. For ATAD2, an initial fragment screen was used to ligand the bromodomain [58]. Inhibitors for the bromodomain and ATPase domain have since been further developed, aiding in function of the individual domains and elucidating its function in breast cancer models [124, 125, 126]. There are also several inhibitors for the bromodomain of BRPF, where inhibition is known to impair osteoclast differentiation [127, 128]. Other inhibitors for the Class IV bromodomains are often non‐selective and inhibit several domains, such as NI‐57, which inhibits BRPF1, BRPF2(1), and BRPF3 with IC_50_'s of 3.1, 4.6, and 150 nM, respectively [129]. Other examples of pan‐selectivity include dual‐inhibitors that target Class IV bromodomains and HDAC8 [130]. With various potent inhibitors for Class I, II, III, and IV bromodomains, several medicinal chemistry trends have arisen and have been discussed elsewhere [131, 132, 133]. Reoccurring functional motifs include methyl isoxazoles, 1,2,4‐triazoles, 1,2‐substituted imidazoles, and pyridinones cores.

## 5 Selective Inhibition for BET Bromodomains

### 5.1 Pan‐BET Inhibitors

**FIGURE 6:**
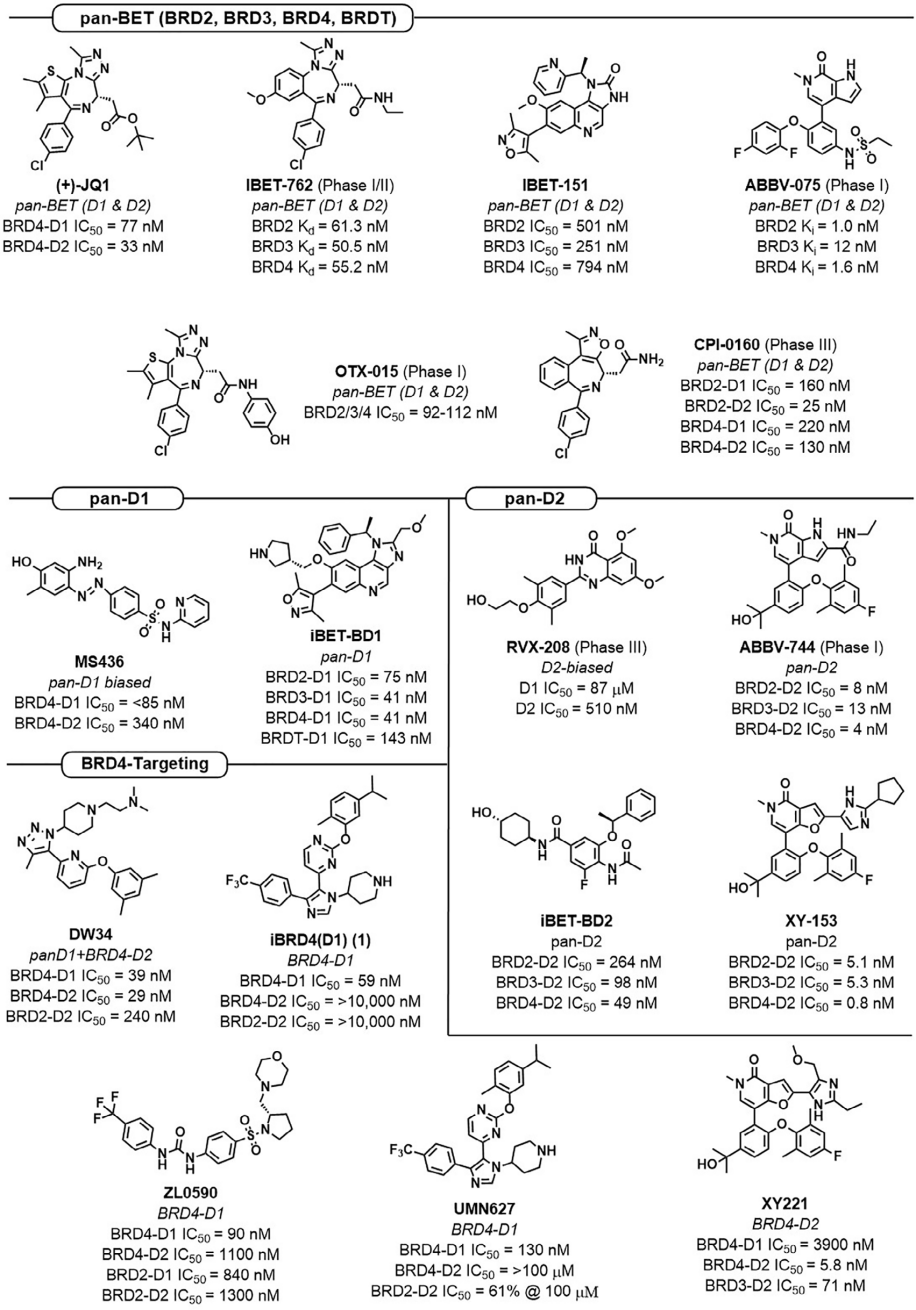
Structures of representative BET inhibitors with varying selectivity. Reported affinities of inhibitors that represent selectivity to their respective bromodomains are also shown. Affinities reported range from a variety of assay formats, and caution should be used when directly comparing absolute affinities across multiple assay formats.

The BET family of bromodomain‐containing proteins (Class II) has been the most heavily investigated given their strong phenotypic response and overexpression in disease [99]. Initially, this family of bromodomain‐containing proteins was investigated for their role in inflammation [134], and concurrently emerged as a key driver in cancer [135]. Potent oncogenic effects were also demonstrated in early work through BRD4‐driven NUT‐midline carcinoma with (+)‐JQ1, a pan‐BET inhibitor (Figure 6) [53, 136]. Further work by Picaud et al. re‐evaluated the role of BETs as master regulatory proteins in leukemia through pan‐bromodomain inhibition with bromosporine (Figure 5) [100]. The widespread use of pan‐BET inhibitors includes multiple disease indications, including diabetes, infectious, and cardiovascular disease [137, 138, 139]. Clinically, however, pan‐BET inhibition has led to gastrointestinal issues and thrombocytopenia, limiting therapeutic progress targeting the BET bromodomains [94, 140]. Despite the dose‐limiting toxicity, given BRD4's importance at super enhancer regions and nuclear condensates to drive downstream transcription [137, 141, 142], BRD4 and the other BET bromodomains remain therapeutically important as potential epigenetic therapeutic targets and disease biomarkers.

Since the discovery of (+)‐JQ1 and IBET‐762 in 2010 [53, 134], multiple BET inhibitors have been reported with varying selectivity profiles, where the field has generally trended toward selectively targeting either BRD4 or the D2 bromodomains for improving tolerability. This progression in selectivity was primarily to combat BET‐mediated toxicity concerns, where other pan‐BET inhibitors had similar toxicity profiles in clinical studies [143]. However, each member of the BET family has been implicated in a variety of diseases, where genetic knockdown experiments support differential effects for each of the BET proteins in varying disease indications [139, 144, 145]. This includes the testis‐specific BRDT, which was overexpressed in some lung and esophageal cancers despite being largely expressed in the testis in normal tissue [146]. Given the diverse disease indications associated with each member of the BET family and their seemingly co‐regulatory functions in controlling gene expression, it stands that targeting individual domains or BET proteins may be beneficial for developing therapeutic approaches. Here, we examine the biological consequences of differentially selective BET inhibitors and targeted protein degraders with a focus on cancer and inflammation models. The efficacious effects of pan‐BET inhibition in cancer have been well established, particularly in NUT midline carcinoma. NUT midline carcinoma is a rare‐but‐aggressive form of cancer that relies on a BRD4‐NUT fusion oncoprotein and is driven via c‐Myc [147]. As such, pan‐BET inhibition (e.g., OTX‐015 and (+)‐JQ1) was particularly efficacious, targeting BRD4‐NUT fusions and displacing this protein from chromatin [148, 149]. While the efficacy of pan‐BET inhibition was reduced in solid tumors [149], cellular and in vivo investigations revealed potential efficacy in hematological malignancies, such as AML and multiple myeloma [150, 151]. Indeed, the importance of Class II bromodomain‐containing proteins remains of interest for therapeutic development.

### 5.2 Pan‐Domain Inhibitors (Pan‐D1, Pan‐D2)

Early examples to address the BET‐mediated toxicity of pan‐BET inhibitors shifted toward selectivity within the BET family. One prominent strategy for gaining selectivity within the BET family relies on targeting the few non‐conserved residues, such as the histidine/aspartate switch available (Asp144 in BRD4‐D1, His437 for BRD4‐ D2) [89, 152]. Early examples of these inhibitors include MS436, which showed modest selectivity toward BRD4‐D1 (*K*_i_ < 85 nM for BRD4‐D1, 340 nM for BRD4‐D2, Figure 6). However, MS436 displayed similar potency toward both bromodomains of BRD3 (*K*_i_ = 100 nM and 140 nM for D1 and D2, respectively) [153]. The same year, RVX‐208 was reported as a D2‐biased inhibitior, which showed significant efficacy toward inflammation [154, 155]. Seminal work by Gilan et al. [89] developed domain selective inhibitors, iBET‐BD1 and iBET‐BD2 (pan‐D1 and pan‐D2 inhibitors, respectively), leading to a significant breakthrough in the development of domain‐selective inhibitors within the BET family. These inhibitors selectively target four of the eight BET bromodomains with nanomolar potency (the four D1 BET bromodomains for iBET‐BD1, the four D2 BET bromodomains for iBET‐BD2). Through these selective inhibitors, iBET‐BD1 demonstrated that pan‐D1 inhibition phenocopied pan‐BET approaches in a variety of cellular cancer models [89]. Similarly, the pan‐D2 inhibitor, ABBV‐744, was efficacious in preclinical models of prostate cancer and AML [156, 157], suggesting that partial inhibition of the BET bromodomains may be an alternative solution to maintain efficacy in cancer while subverting BET‐mediated toxicities. In inflammation, however, the role of the D2 bromodomains within the BET family remains contested, as pan‐D2 inhibition was sufficient to reduce inflammation in some models, whereas other models showed the most dramatic effect with BRD4 knockdown [158] or a combination of BET proteins [139, 144]. Additionally, a series of BRD4‐D1 selective inhibitors were effective at reducing downstream inflammatory cytokines in liver inflammation models, similar to other reports showcasing the efficacy of D1‐targeting approaches [97, 139, 159]. Varying effects are observed in inflammation between the chemical inhibition of the BET bromodomains versus transcriptional knockdown, suggesting that pan‐BET inhibition may not be required in different models of inflammation due to the auxiliary domains of the BET proteins and additional protein–protein interactions [144, 158].

### 5.3 BRD4‐Selective Inhibitors

To further probe the individual functions of BET bromodomains, more selective ligands have been developed to probe BRD4 with domain selectivity. This includes UMN627, a BRD4‐D1 selective inhibitor, which achieves selectivity by the selective targeting of Asp144 and the displacement of structured waters, and was efficacious in liver inflammation models (Figure 6). However, in the context of MM.1S cancer models, only modest activity was observed [57, 160]. Further optimization of this scaffold gave iBRD4(D1), which had a higher affinity toward BRD4‐D1 and a greater selectivity over other BET bromodomains compared to its predecessor [161]. Our lab has previously reported DW34, which has the unique selectivity profile of inhibiting the D1 bromodomains in addition to BRD4‐D2 (pan‐D1+BRD4‐D2) [63, 152]. This compound mimics pan‐BET inhibition in reducing c‐Myc expression in MM.1S while also showing comparable efficacy in liver inflammation models. However, DW34 still showed a significant preclinical thrombocytopenia risk, likely due to the inhibition of the D1 bromodomains [63]. Indeed, examination of BRD4‐D1 selective inhibitors, such as UMN627 and iBRD4(D1) [160, 161], in addition to the recently developed BRD4‐D2 selective inhibitor, XY221 [162], can help determine the mechanism of BRD4 over other BET bromodomains. Other motifs utilize dual‐domain approaches to engage both domains of BET proteins simultaneously; though, this approach was not selective for BRD4 over the other BET proteins [163].

Additional pharmacophores were also reported as BRD4‐D1 selective inhibitors, such as ZL0454 and ZL0590, the latter of which is hypothesized to engage the bromodomain from an allosteric site [164, 165]. These compounds were initially shown to be efficacious at reducing BRD4‐mediated acute airway inflammation. The same group also reported two compounds that relied on the RVX‐208 scaffold, ZL0516 and ZL0969. Compound ZL0516 was initially discovered through scaffold‐hopping efforts, where it was biased toward BRD4‐D1 and moderately efficacious against poly‐(I:C)‐induced inflammation through the downregulation of IL‐6 [166]. Recent optimization of this scaffold afforded the next‐generation ZL0969, which was similarly efficacious in regulating inflammation in the lung [167]. Interestingly, ZL0969 is reported to be biased toward BRD4‐D1 and BRD4‐D2 (∼10−15 fold selective over other BET bromodomains), despite the original D2‐biased selectivity profile of the RVX‐208 parent scaffold. While BRD4‐D1 targeting molecules are continuing to be developed and tuned for cellular environments, the development of an inhibitor targeting the second bromodomain of BRD4 (BRD4‐D2) has also emerged. In this case, XY221 was reported to have 66–142‐fold selectivity for BRD4‐D2 compared to the other BET D2s, where selectivity arises from capitalizing on interactions within the BC‐channel [162]. Similar to the efficacy of pan‐D2 inhibitors, XY221 showed significant anti‐proliferative effects against MV4−11 cells through reduction of c‐Myc transcription. This anti‐proliferative activity was reported to be cell‐line specific, where other c‐Myc driven cancers, such as MM.1S, were up to 10‐fold less responsive to BRD4‐D2 selective inhibition. Indeed, given the advancements of BRD4‐selective and domain‐selective approaches for inhibiting BET bromodomains, future work should prioritize the mechanistic investigation of individual BET bromodomains in comparison to less selective approaches. In doing so, a more complete understanding of BET‐driven disease can be obtained, especially in the context of inflammation.

## 6 The Biological Impact of Differentially Selective BET Inhibition

### 6.1 Pan‐D1 Inhibition

Seminal work by Gilan et al. [89] led to the development of iBET‐BD1, a pan‐D1 inhibitor with the highest degree of selectivity to date. Inhibitor iBET‐BD1 was able to phenocopy pan‐BET inhibition in their transcription profiles. Further, in the AML cell line MOLM‐13, pan‐D1 inhibition was comparable to pan‐BET inhibition in terms of reducing cellular proliferation and inducing apoptosis (Table 1). Additional cell cycle analysis revealed pan‐D1 inhibition was mechanistically similar to IBET‐151 in MV4−11 cells, as well as increasing the overall survival in in vivo leukemic settings. Interestingly, the highly selective pan‐D2 inhibitor, iBET‐BD2, failed to significantly influence these factors, highlighting the role of the D1 bromodomains in regulating Myc‐driven cancers. In inflammation, the early pan‐D1 inhibitor MS436 was shown to be effective at reducing NO‐synthesis in LPS‐stimulated systems, though pan‐D2 inhibition had similar effects [153]. This effect was similar to results observed by Gilan et al., in which case both pan‐D1 and pan‐D2 inhibition were effective at reducing the localization of BET proteins after INFγ‐induced inflammation.

**TABLE 1:** Differing biological effects between selective BET inhibition profiles.

Selectivity	Biological effect	Citations
Pan‐BET	• Potent anti‐proliferative and anti‐cancer effects in a variety of different cell models (AML, NUT‐midline carcinoma, breast cancer, high grade gliomas, others)	[72, 147, 149, 150, 151]
	• Anti‐inflammatory effects in several inflammation models (NF‐kB and INF‐γ driven)	[89, 153, 158, 168]
	• Significant risk for BET‐mediated toxicities that have hindered clinical development (anemia, thrombocytopenia, and gastrointestinal effects)	[63, 90, 157]
Pan‐D1	• Transcriptionally phenocopies pan‐BET inhibition, comparable anti‐proliferative effects to pan‐BET inhibition in cancer models. • Differential effects on c‐Myc with less‐selective inhibitors	[89, 169, 170] [171]
	• Anti‐inflammatory effects in diabetes model through BRD4‐p65 disruption.	[139]
Pan‐D2	• Model‐specific effects on cell proliferation cancer models (MV4−11, AML cell lines, prostate cancer lines)	[154, 157, 172]
	• Differential impact on N‐Myc in neuroblastoma models compared to pan‐BET inhibition • Differential impact on gene expression, regulating less genes compared to pan‐D1 inhibition	[89, 173]
	• Anti‐inflammatory effects are also model‐dependent, shown in INF‐γ stimulated K562, but not in liver inflammation or diabetes models	[63, 89, 139]
	• Better tolerated in terms of BET‐related toxicities, where D2‐selective inhibitors show less potential for thrombocytopenia in pre‐clinical models	[174, 175]
BRD4‐D1	• Lack of reduction in c‐Myc in MM.1S, though still underexplored in cancer	[160]
	• Anti‐inflammatory effects both in liver inflammation models through the displacement at super‐enhancer regions • Reduced poly(I:C)‐induced inflammation through BRD4‐D1	[63, 97, 165]
	• Dramatic reduction in pre‐clinical thrombocytopenia risk	[63]
BRD4‐D2	• Selective anti‐proliferative properties for MV4‐11	[162]
	• Remains underexplored in various inflammation models and for distinct mechanistic effects due to limited chemical probes	
BRD4‐D1 & BRD4‐D2	• No known information, co‐dosing or genetic editing experiments are needed to decipher full function.	

Even with the development of early pan‐D1 inhibitors, the differential effects of D1 versus D2 versus pan‐BET inhibition on cell differentiation became apparent. A moderate‐affinity pan‐D1 inhibitor, Olinone, induced cell differentiation in the case of myelin‐forming oligodendrocytes [176]. This was the opposite effect compared to pan‐BET inhibitors, which prevented their degradation. Treatment with D2‐biased inhibitor RVX‐208 had no signifying effect on cell differentiation. Counterintuitive effects were also observed with Compound 1, originally reported by Raux et al. in a mid‐throughput screen [171]. This compound either stabilized or promoted the expression of c‐Myc at 1 μM, where c‐Myc was eventually downregulated at higher concentrations that eroded Compound 1's selectivity. The observed impact on cell differentiation and c‐Myc expression with Olinone and Compound 1, respectively, may have been due to cellular off‐targets and their micromolar affinity toward D1 domains. As more potent and more selective inhibitors were developed, such as either iBET‐BD1 (IC_50_ = 40–140 nM) or GSK789 (*K*_d_ = 20 nM), the anti‐proliferative activities of pan‐BET and pan‐D1 inhibitors converged. For GSK789, similar efficacy toward cell viability were observed compared to IBET‐151 in MV4−11, HL–60, and THP–1 cells [169]. It remains to be seen whether pan‐D1 inhibition is better tolerated with regards to BET‐mediated toxicity.

In general, pan‐D1 approaches continue to be important part of phenocopying pan‐BET inhibition in cancer models, where they are still being explored in inflammation settings. For mechanistic analysis, pan‐D1 inhibitors can serve as useful tools to explore variations in gene expression when paired with other selective inhibitors (pan‐D2). As BET inhibitors continue to develop; however, it is important to note the role of concentration in cellular studies when drawing conclusions based on BET selectively when interpreting molecular mechanisms for regulating gene expression. From a clinical perspective, pan‐D1 inhibitors are likely to suffer the same fate as pan‐BET inhibitors in the past due to dose‐limiting toxicities. This is further supported by the lack of new clinical data with these selective compounds. As these compounds continue to develop, more protein‐specific compounds (i.e., iBRD4(D1) or XY‐153) may be more likely to be successful in developing novel therapeutic approaches to the BET family.

### 6.2 Pan‐D2 Inhibition

Diverging from their pan‐D1 counterparts, targeting the second bromodomains of the BET family (D2s) has had mixed effects toward cell proliferation and gene regulation. RVX‐208, a pan‐D2 biased inhibitor has minimal effect on c‐Myc expression in cells, impacts ∼15‐fold less genes compared to (+)‐JQ1 (46 and >700 genes, respectively), and requires much higher concentrations to displace BRD4 from chromatin (Table 1). Taken together, the lack of response from RVX‐208 casts doubt on solely BET‐dependent mechanisms [154, 172]. ABBV‐744 showed weak anti‐proliferative activities in a panel of cancer cell lines aside from select AML and androgen‐receptor positive cell lines [157, 175]. Other pan‐D2 inhibitors have also shown similar cell‐line selectivity for MV4−11 cells [177]. In these models; however, ABBV‐744 was as efficacious as pan‐BET inhibitor ABBV‐075 both at a cellular and in vivo level. This difference suggests the broader safety profile of targeting the D2 bromodomains in terms of off‐tissue toxicity. However, Gilan et al. suggest that the efficacy of ABBV‐744 is a result of its slightly increased activity toward BRD4‐D1, potentially explaining the varied anti‐proliferative effects between ABBV‐744 and iBET‐BD2 (83‐fold and >130 fold selective for D2, respectively) [89]. In neuroblastoma, where BRD4 controls the expression of the oncogenic MYCN, pan‐D2 inhibitor SJ432 was particularly efficacious [173]. Here, differential effects on gene expression were observed escalating the dose of SJ432, eventually converging onto pan‐BET effects as selectivity eroded. This study highlights time‐dependent effects on MYC expression, where (+)‐JQ1 caused a rebound of BRD4 levels in SK‐N‐AS cells, not observed from SJ432 treatment. SJ432 also had a more rapid and efficacious effect at reducing MYCN levels. In summary, D2 inhibitors may provide a more tolerable approach to target BRD4 but their efficacy is highly disease dependent. Thus, these compounds serve to be both useful chemical probes for the same reasons as pan‐D1 inhibitors. Their therapeutic potential, however, is trending toward being limited to very specific disease states, or when combined with other treatments in clinical trials. Current findings support the development of probes or modalities that target BRD4‐D1 and ‐D2, where heterobifunctional molecules may bridge these gaps in efficacy.

## 7 Clinical Efforts Toward Bromodomain‐Containing Proteins

### 7.1 BET‐Targeting Therapies

**FIGURE 7:**
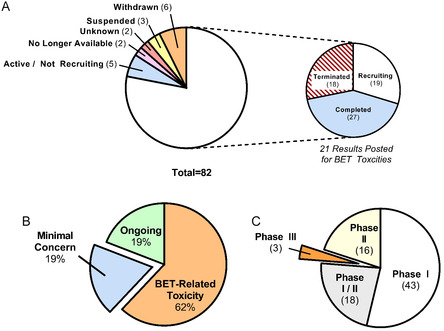
Analysis of BET‐targeting inhibitors in clinical trials. (A) Current status of clinical trials targeting BET proteins by status analyzed (82 total), with completed (blue, 27 entries), recruiting (white, 19 trials), and terminated (red striped, 18 entries) shown on the right. All data was obtained from clinicaltrials.gov. Number of trials analyzed shown in parentheses. (B) Portion of ongoing (19%, green) or completed clinical trials that report significant BET‐related toxicities by compound (62%, orange), minimal concern (19%, blue) towards BET‐related toxicities. Data collected as of May 2026. (C) Portion of BET‐targeting therapies in each phase of clinical trials, including combination therapies. Phase III compounds are limited (either in monotherapies or combination therapies, shown in orange) with only 3 trials progressing to Phase III. Number of trials analyzed shown in parentheses.

Given the dysregulation of BET proteins in human malignancies and their important role in gene transcription and cell‐cycle progression, the BET family of proteins has been the target of drug discovery efforts since their inception [178]. Likewise, the majority of bromodomain inhibitors that have progressed to clinical trials target BET proteins due to their strong phenotypic response. The BET family members, BRD4, BRD3, and BRD2, are ubiquitously expressed in healthy tissues, where BRDT is primarily expressed in the testes (though each of these proteins has also been implicated in a variety of cancers) [135, 146, 179, 180]. The regulatory role of BET proteins in healthy tissues has been a primary cause for concern in developing BET‐targeting therapies, leading to the development of gastrointestinal issues and thrombocytopenia through pan‐BET inhibition [94, 140]. Further, the in vivo silencing of BRD4 revealed a range of different toxicities compared to (+)‐JQ1 treatment, including the loss of intestinal stems that were not present with pan‐BET inhibition [181]. To better address the current status of BET‐related clinical trials, we analyzed active and non‐active clinical trial results (82 total) to assess both BET‐mediated toxicities as well as trial status (Figure 7). The toxicities associated with BET inhibition have naturally attenuated their use as therapeutics, with 62% of BET inhibitors (Figure 7B) in clinical trials noting some degree of BET‐mediated toxicity (defined here by either Grade 3/4 gastrointestinal distress, Grade 2 thrombocytopenia, or >20% of patients experiencing dose‐limiting toxicities). Recent mechanistic studies have linked the inhibition of BET proteins with the downregulation of GATA1 in both preclinical and clinical settings, a hematopoietic transcription factor. In turn, this alters proteins involved in megakaryopoiesis and thrombopoiesis, resulting in undesired clinical outcomes [94, 182]. These are also supported by related clinical work [183] in addition to the movement towards more selective inhibition. Seminal work with BRD4‐knockdown in transgenic mouse models suggest that sustained BRD4 inhibition has dramatic effects on multiple tissues [181]. Whether different therapeutic approaches, such as PROTACs, can solve these issues with targeting BRD4 directly remains an important question warranting investigation.

**FIGURE 8:**
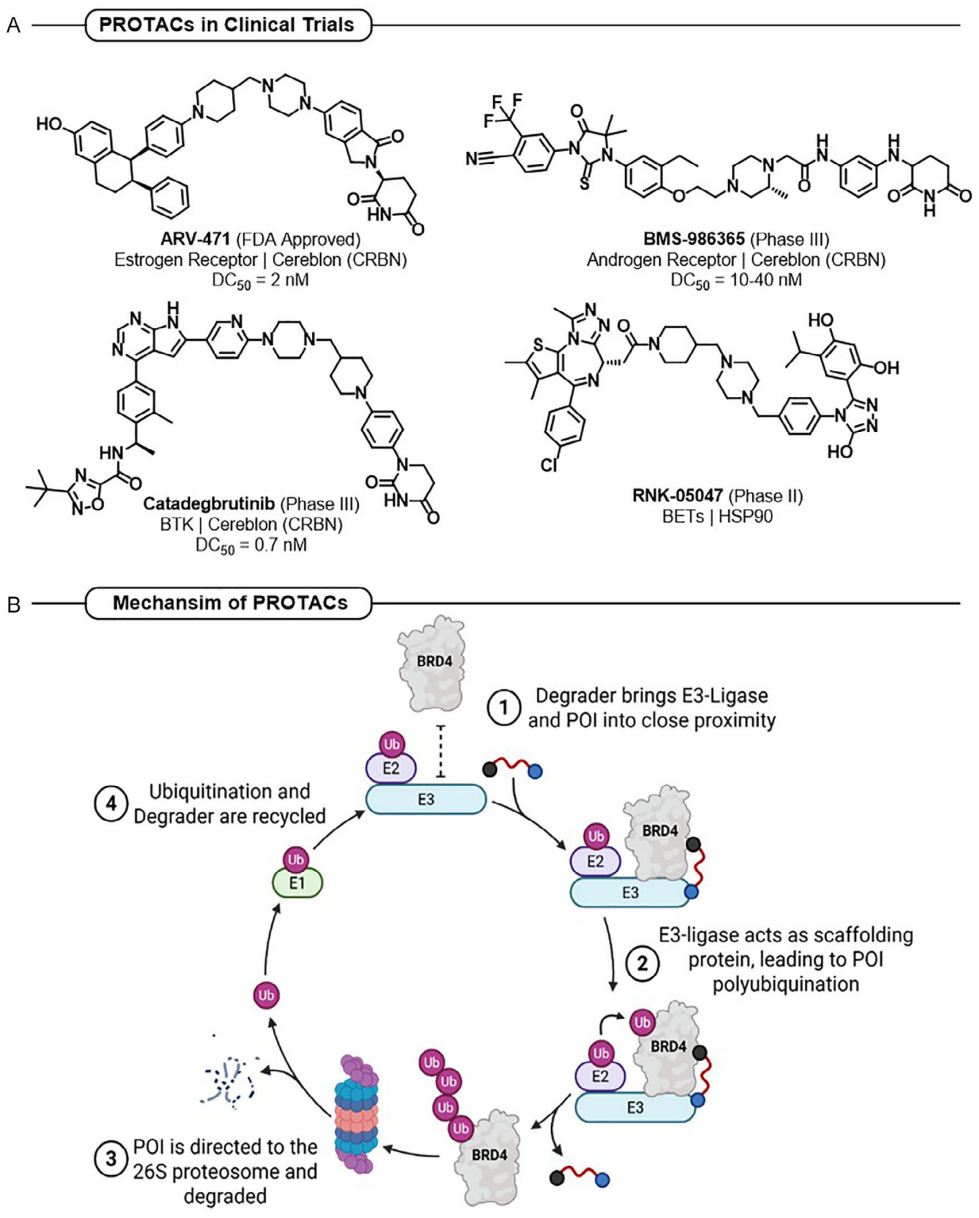
Mechanisms and examples of PROTACs in clinical trials. (A) Examples of PROTACs in clinical trials with protein targets and cellular characterization (as available). PROTACs are oriented so that the protein‐of‐interest ligand is on the left, with the E3‐ligase ligand on the right. (B) General cellular mechanism of PRTOACs with BRD4 as a model system. PROTACs operate through auxiliary scaffolding proteins to facilitate protein degradation, shown for BRD4 (gray), leading to the ternary complex between BRD4 and an E3‐ligase (E3, blue). E3 acts as scaffolding protein for E2, and eventually the polyubiquitination of BRD4 and its degradation through the proteosome.

Other clinical efforts are either ongoing or involve more selective inhibitors in order to reduce BET‐mediated toxicities, such as ABBV‐711 (pan‐D2, NCT04454658) or RVX‐208 (D2‐biased, several clinical trials). Other alternatives include combination therapies to achieve the desired efficacy or to optimize dosing schedules of pan‐BET inhibitors [143]. Due to the substantial rate of BET‐mediated toxicities, only 2 compounds have progressed to Phase III trials through three separate trials (NCT06401356, NCT02586155, NCT04603495) shown in Figure 7C. These compounds, CPI‐0610 and RVX‐208, were investigated for treatment against myelofibrosis and cardiovascular disease, respectively. The efficacy of more selective BET inhibitors (i.e., ABBV‐744) [175] remains unclear in late‐stage settings, though preclinical models of thrombocytopenia are encouraging [157].

Despite the concerns of BET‐mediated toxicities, the efficacy of BET‐targeting treatments has inspired the progression of BET inhibitors as therapeutics for both monotherapies and combinative approaches. The use of BET inhibitors in clinical efforts has largely been in the treatment of cancer, with multiple Phase I/II trials in progress. Preclinical evaluation of pan‐BET inhibition in multiple myeloma led to hopes for achieving a clinical response in this disease indication [140, 178]. In one of these trials, treatment with OTX‐015 in patients with multiple myeloma displayed responsiveness (*n* = 45), whereas only two patients with c‐Myc positive diffuse large B‐cell lymphoma met the response criteria [184]. The lack of significant clinical outcomes from these trials may partially be attributed to dose‐limiting toxicities, highlighting an important need for pharmacodynamic investigations into pan‐BET inhibitors to guide dose‐escalation studies. Similarly, the pan‐BET inhibitor IBET‐762 was evaluated in NUT carcinoma in Phase I/II studies as a monotherapy, though a similar spectrum of adverse effects was observed related to pan‐BET inhibition [185]. Due to these adverse effects, pan‐BET inhibitors are currently being investigated as combination therapies, where CPI‐0610 is being used in combination with Janus Kinase 1 (JAK1) inhibitors for the treatment of myelofibrosis (Phase III, NCT04603495).

Efforts from AbbVie include both pan‐BET and pan‐D2 inhibitors, with the development of ABBV‐075 and ABBV‐744, respectively. The use of pan‐BET inhibitor ABBV‐075 as a monotherapy (Phase I, NCT03360006) is currently in progress for the treatment of AML. Additional investigation into ABBV‐075 included evaluation of solid tumors, where ABBV‐075 treatment in combination with a Bcl‐2 inhibitor showed 43% stable disease indications with similar reports of dose‐limiting toxicity [186]. These results emphasize the need for more selective bromodomain inhibitors for the BET family, where BRD4‐selective approaches may reduce the toxicities associated with BET inhibition. ABB‐744, a pan‐D2 inhibitor, is currently being investigated in Phase I trials for the treatment of AML and myelofibrosis (NCT03360006, NCT04454658). While it remains unclear whether these more selective inhibitors reduce clinical dose‐limiting toxicities, pre‐clinical efforts suggest that ABBV‐744 is well‐tolerated for thrombocytopenia risks while showing significant antiproliferative effects [157]. The use of more selective BET inhibitors is only recently being investigated in clinical settings; the use of selective BET inhibitors in pre‐clinical settings gives hope for overcoming toxicity challenges in targeting this important class of epigenetic regulators. BRD4‐selective approaches may provide some progress. Such a strategy is supported by Lamonica et al. [94] who show that BRD2 and BRD3 inhibition contributes to these toxicities. We have also shown improved safety of BRD4‐D1‐selective inhibitors, which were >50‐fold more tolerated in a preclinical thrombocytopenia model compared to ABBV‐075 [63]. Indeed, as other BRD4‐targeting approaches (such as PROTACs, which are only now progressing to clinical trials) progress in drug‐development campaigns, it is crucial to understand the role of individual BET bromodomains to inform therapeutic efforts.

### 7.2 Non‐BET Inhibitors in Clinical Trials

While targeting BET proteins through chemical inhibition and PROTAC approaches is well‐described, targeting of non‐BET bromodomain‐containing proteins is now progressing into the clinic. These consist of six primary therapeutic targets: BRD9, CBP/p300, SMARCA2/4, and GCN5 (Table 2). For BRD9, the two therapeutic modalities chosen included FHD‐609 (NCT04965753) and CFT8634 (NCT05355753); both compounds were BRD9‐targeting PROTACs. Despite the improved efficacy of PROTACs for BRD9 over their inhibitor counterparts (discussed above in Section 4), both trials have now been terminated, with CFT8634 showing insufficient efficacy for heavily pre‐treated synovial sarcoma. Therapeutic strategies targeting CBP/p300 have relied on the inhibition of HAT domains, rather than targeting the bromodomain (NCT07400913, NCT05488548). While one of these trials is currently recruiting, it remains to be seen if combination approaches (through inhibition of the bromodomain and HAT domain, or through other mechanisms) or PROTAC‐based approaches provide meaningful therapeutic benefit. Both inhibitors (e.g., LY4050784, Phase I) and PROTACs for SMARCA2/4 are currently being investigated in clinical settings against a variety of cancers. The majority of these are currently recruiting for Phase I and Phase II (4 of 7 total). A PROTAC for SMARCA2 includes QLH12016, which is currently being investigated for treatment of advanced prostate cancer (Phase I, NCT07104110). Another examples of early clinical progress is AUTX‐703, which targets GCN5 for the treatment of refractory AML (Phase I, NCT06846606) [187, 188]. Notably, PROTACs present in clinical settings utilize heterocyclic linkers, such as those employed by Arvinas, though aliphatic and PEG‐based linkers predominated early PROTAC development [189]. These PROTAC approaches predominate therapeutic approaches for non‐BET bromodomain‐containing proteins compared to small‐molecule inhibition found in the majority of BET clinical settings. This suggests an interesting paradigm in targeting bromodomain‐containing proteins found in large transcriptional regulation complexes (e.g., SMARCA2/4 in SWI/SNF complexes) and warrants further investigation into these spaces.

**TABLE 2:** Summary of clinical trials targeting non‐BET bromodomain‐containing proteins.

Target	Compound	Phase	Indication	Identifier	Status
CBP/p300 (Class III)	TT125‐802	Phase I	Advanced solid tumors	NCT06403436	Active, not recruiting
	EP31670	Phase I	NUT carcinoma, castrate‐resistant prostate cancer, others	NCT05488548	Recruiting
BRD9 (Class IV)	CFT8634	Phase I	Synovial sarcoma, soft tissue sarcoma	NCT05355753	Terminated
	FHD‐609	Phase I	Advanced synovial sarcoma	NCT04965753	Terminated
SMARCA 2/4 (Class VIII)	PLX‐61 639	Phase I	Esophageal squamous cell carcinoma	NCT07284186	Recruiting
	QLH12016, abiraterone, enzalutamide	Phase I/II	Prostatic neoplasms	NCT07104110	Not yet recruiting
	PRT3789, pembrolizumab	Phase II	Advanced solid tumors	NCT06682806	Terminated
	LY4050784, cisplatin, pembrolizumab, Paclitaxel	Phase I	Metastatic solid tumors	NCT06561685	Recruiting
	PRT7732	Phase I	Advanced solid tumors, Advanced metastatic tumors	NCT06560645	Terminated
GCN5 (Class I)	AUTX‐703	Phase I	Relapsed AML, Refratory AML	NCT06846606	Active, not recruiting

## 8 PROTACs as an Emerging Therapeutic Modality for the BET Family of Proteins

### 8.1 PROTAC Development, Mechanisms, and Early Strategies

Targeted protein degradation is an emerging therapeutic modality that relies on the E3 ubiquitin‐protease system to initiate protein degradation. Early strategies emerged in proteolysis targeting chimeras (PROTACs), which use three components to initiate degradation of a target protein: a target binding moiety, a linker region, and an E3‐binding moiety. In doing so, these heterobifunctional molecules bring the target protein and the E3 ligase into close proximity (Figure 8). The E3 ligase then acts as a scaffolding protein for E2 ubiquitination machinery, leading to the polyubiquitination and subsequent degradation of the target protein through the 26S proteosome [190, 191]. PROTACs operate differently compared to small‐molecule inhibition, as a single PROTAC molecule can act sub‐stoichiometrically, enabling lower doses and complete degradation of auxiliary binding domains. This is particularly relevant to BET‐targeting PROTACs, as lower dosing regimens may lead to fewer dose‐limiting toxicities in clinical settings. However, this comes with significant optimization costs, as PROTACs are inherently beyond Rule‐of‐5 molecules, greatly increasing their molecular complexity and size for biological applications [190, 192]. This also imbues cellular challenges with cellular permeability and metabolism, which are better understood for small‐molecule approaches.

**FIGURE 9:**
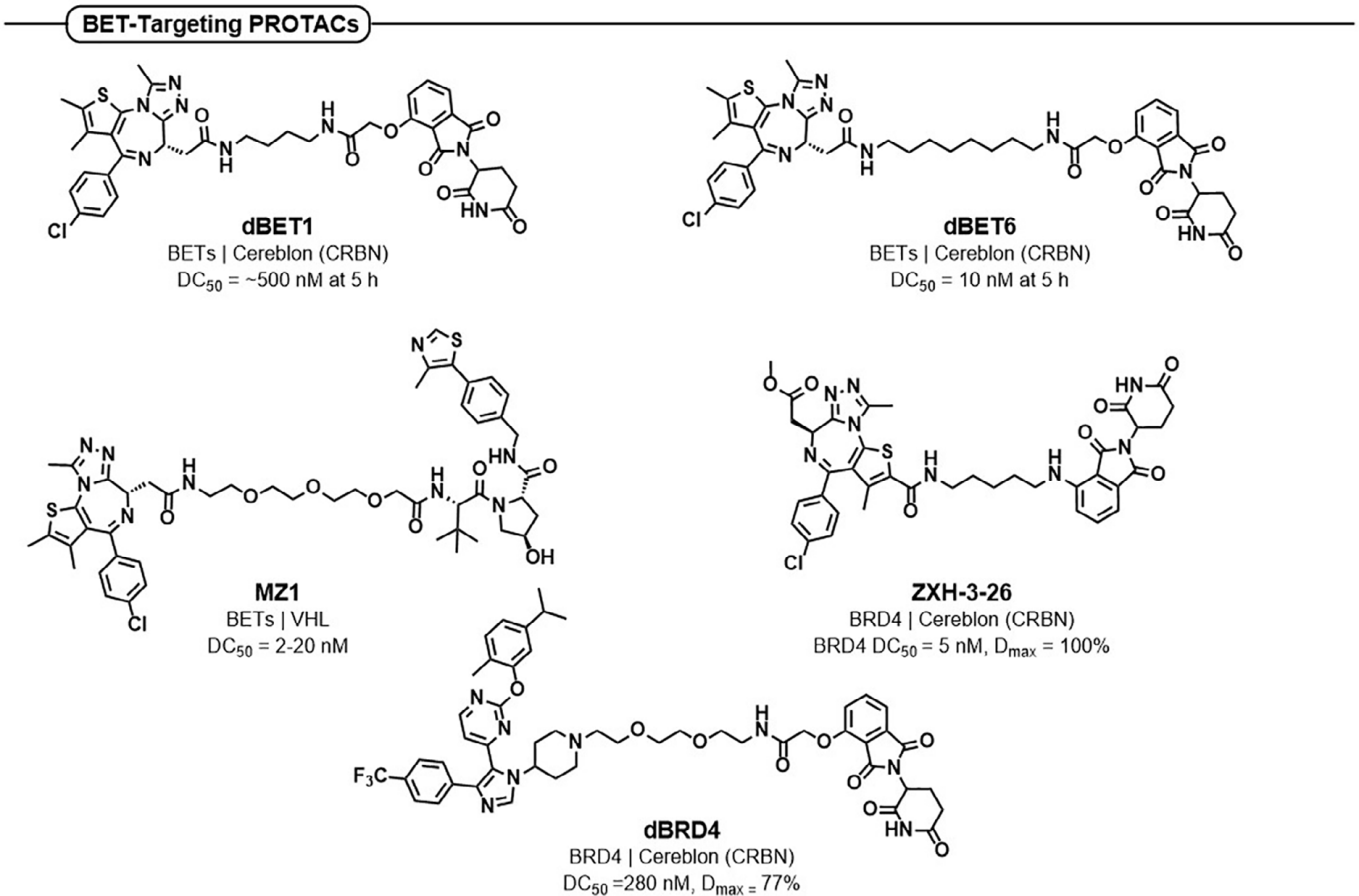
Structures, potency, and selectivity of representative BET‐targeting PROTACs. Target proteins and E3 ligases are listed for each compound. Compound structures are arranged so that the target‐binding ligand is oriented on the left and the E3‐ligase biding ligand is oriented on the right of each structure. DC_50_ values are listed where available. Notably, all BET‐targeting PROTACs use (+)‐JQ1 as a parent BET ligand aside from dBRD4, which uses iBRD4(D1) to incur additional selectivity.

The first PROTAC was reported by Sakamoto et al. [193] in 2001, which initially used a peptide‐based ligand to recruit E3 ligase, Ik1β. These compounds initially struggled with cell permeability, which was then overcome to degrade the androgen receptor in cellular contexts. This was quickly followed by the report of cellular PROTACs, PROTAC‐2, in 2003, which was active in cells [194]. In 2008, the first small‐molecule PROTACs were also described by the Crews lab, using a Ik1β ligand to promote the degradation of the androgen receptor [195]. Though these early approaches used peptides as E3 ligase ligands, which have remained limited due to its poor degradation efficiency and cell permeability challenges. Rather, the E3 ligases cereblon (CRBN) and Von Hippel‐Lindau tumor suppressor (VHL) have been the most widely used E3 ligase targets for PROTAC development, likely due to their synthetic accessibility, well‐characterized and specific binding modes, and physiochemical properties (Figures 8 and 9) [196]. Nearly a decade after first reports of PROTACs, small molecule ligands, thalidomide and lenalidomide, were reported to bind to E3 ligase CRBN, where VHL ligands were described shortly afterwards [197, 198]. These were then coopted into the PROTAC heterobifunctional scaffold, leading to the degradation of the BET family proteins in cellular context through the recruitment of either the CRBN with dBET1 or the Von Hippel‐Lindau tumor suppressor (VHL) with MZ1 (Figure 9) [199, 200]. Since the development of this targeted protein degradation modality, PROTACs have been described for a myriad of disease‐related targets, including targeting PD‐L1 [201], BET bromodomain‐containing proteins [202], and anaplastic lymphoma kinase (ALK) [203]. Several disease states have also been targeted through PROTACs over the past 20 years, demonstrating the importance of this emerging therapeutic modality for a variety of malignancies [204, 205, 206, 207, 208, 209]. The importance of PROTAC development for therapeutic developments is best showcased by clinical data; there are >30 current PROTACs in clinical trials, with ARV‐471 being the first PROTAC to receive FDA approval (Figure 8) [204, 210].

Despite the advances in E3‐ligase ligands, the vast majority of PROTACs have relied on recruiting either CRBN or VHL. However, in efforts to achieve either tissue selectivity, desirable drug‐like properties, or differential expression profiles, significant effort has been placed in developing ligands targeting different E3 ligases or exploring alternative degradation mechanisms [190]. Interestingly, attempts to engage multiple bromodomains within the BET family through trivalent mechanisms revealed that VHL‐recruiting compounds were more efficacious compared to trivalent CRBN‐recruiting compounds, highlighting the cooperative mechanisms within varying E3 ligases [211]. One approach in targeting alternate E3 ligases includes through covalent‐glue mechanisms by attaching functional motifs to inhibitors (including (+)‐JQ1) to recruit either RNF126 or DCAF16 [212, 213]. Other E3 ligases that have been explored for BET degradation include KEAP1 [214], MDM2 [215], or HSP90 [216]. A recent example uses MAGEA11 as an tissue‐selective E3‐ligase to selectively degrade BRD4 in both U2OS and KYSE180 cells over healthy cell models [217]. These approaches highlight a primary benefit of expanding on the use of E3 ligases in PROTAC approaches, where tissue‐selectivity can be achieved in combination with high efficacy. Ligands of apoptosis proteins (IAP) have also been explored, leading to the development of targeted protein degraders operating through slightly different mechanisms, deemed SNIPERs [218]. Other types of protein degradation modalities continue to emerge for bromodomain‐containing proteins, including molecular glues [219, 220] and allosteric modulators [221, 222], but remain an emerging field compared to PROTACs. Molecular glues function similarly to PROTACs in their degradation mechanisms but rely on cooperativity between the protein‐of‐interest and the E3‐ligase (rather than connection through a linker motif) in order to induce degradation. Notable molecular glues are continually being developed for bromodomain‐containing proteins to circumvent some drawbacks with PROTACs while maintaining their mode‐of‐action, mainly their molecular size and complexity. Often, molecular glues are quite potent due to their reliance on cooperativity. Recent examples of molecular glues targeting bromodomain‐containing proteins have been developed by Genentech targeting SMARCA2/4 with the FBXO22 E3‐ligase (DC_50_ = 17 pM, *D*_max_ = 95%) [223], and by Amphista Therapeutics targeting BRD9 (DC_50_ = 0.05 nM, *D*_max_ = 88%) through a DCAF16‐mediated electrophilic warhead [224].

### 8.2 BET‐Targeting PROTACs and Their Selectivity

In the context of targeting BET bromodomains, both dBET1 and MZ1 have been used as potent chemical probes to assess BET biology in the contexts of cancer, inflammation, and immune response [180, 225, 226]. These molecules have been able to shed light upon the differences in downstream transcriptional effects when comparing bromodomain inhibition, where BET proteins still retain scaffolding functions, and BET degradation. Generally, BET‐targeting PROTACs have been efficacious in reducing the expression of c‐Myc in cancer models, similar to pan‐BET inhibition [227, 228]. In non‐cancer settings, pan‐BET degradation is also efficacious against neuroinflammation through oxidative stress mechanisms [229]. In vivo treatment of retinal degeneration models with pan‐BET PROTAC, dBET6, had a protective effect against light damage through downstream inhibition of the cGAS‐STING pathway, similar to treatment with (+)‐JQ1 [230]. However, the dBET‐series of PROTACs and MZ1 both described approaches using pan‐BET ligands to recruit the BET proteins, which has the potential for similar BET‐mediated toxicity as their pan‐BET inhibitor counterparts. Though BET degradation through PROTACs have largely phenocopied pan‐BET inhibition in cancer models, the few investigations into inflammation have limited the insight into the biological role of BET proteins within these pathways. Rather, these insights have largely been gained by genetic knockdown, and there remains ample opportunity to investigate BET degradation within this space. This is especially true for comparative studies, which would aid in elucidating the role of allosteric functions in these complex biological settings.

Attempts to avoid BET‐mediated toxicity have involved optimization of the linker region to confer selectivity within the BET family through building cooperativity within the ternary complex. Optimization of the linker region for selectivity was first described by Nowak et al. [231] with the development of ZXH‐3‐26 (Figure 9), an efficacious BRD4‐selective compound. Other alternative approaches include the use of pan‐D2 ligands to avoid thrombocytopenia concerns [180]. While these do achieve selective degradation of BRD4 over other BET proteins, the use of non‐selective ligands also creates a mixed selectivity profile of inhibition and degradation within the BET family. In doing so, these PROTACs potentially confound conclusions on potential biological mechanisms for individual BET proteins and may be indicative of pan‐BET approaches. To address these concerns of mixed selectivity profiles, we developed dBRD4, a BRD4‐selective PROTAC that uses a BRD4‐D1 selective ligand to avoid inhibition of additional BET bromodomains [161]. dBRD4 was had moderate efficacy in degrading BRD4 in MM.1S cells (DC_50_ = 280 nM at 24 h). However, selective degradation of BRD4 had a limited response on c‐Myc expression. Further, BRD4‐selective degradation led to a minor upregulation of BRD2 and BRD3, where the mechanism of these changes remains unclear. Indeed, the development toward BRD4‐selective degradation mirrors that of inhibitor development, where selective approaches may reveal mechanistic differences between individual BET proteins. It is therefore important to understand the role of the individual BET bromodomains or BET proteins in a given disease indication, where the BET family proteins serve as key epigenetic regulators for disease indications.

### 8.3 The Biological Impact of BET Degradation

Pan‐BET or pan‐D1 bromodomain inhibition can displace BET proteins from chromatin in some models, including in the case of nuclear condensates [232]. In these systems, BRD4 is found at super enhancer regions to drive the activation of downstream gene expression through complex networks. Thus, PROTACs targeting the BET bromodomains have been a useful tool for studying BRD4's role in driving activity at these super enhancers and in these condensates due to their role in degrading BRD4 entirely [142, 233]. In terms of biological effects, BRD4 degradation also eliminates the C‐terminal motif (CTM) of BRD4, preventing its engagement with p‐TEFb [179]. As such, pan‐BET degradation is efficacious in a variety of different cancer types, including having effects in primary AML models [234]. The pan‐BET PROTAC, dBET1, was also more effective compared to (+)‐JQ1 in inducing apoptosis in MV4−11 models, where BET degradation was more efficacious over time. Similar effects were observed in vivo, where the next generation PROTAC, dBET6, showed anti‐tumor effects in both hematologic and solid tumors [180, 235, 236]. While both of these compounds use CRBN as an E3 ligase, the VHL‐recruiting PROTAC MZ1 had a surprisingly different response on downstream gene expression. The initial investigation of MZ1 in HeLa cells discover that MZ1 differentially regulates downstream gene expression compared to (+)‐JQ1, particularly in the case of *HEXIM1*, *TYRO3*, and *FAS.* [200] While these PROTACs did somewhat phenocopy siRNA knockdown of BRD4, these initial findings suggest alternative BET mechanisms in HeLa cells. Indeed, BET degradation exhibits larger cell‐line effects. For example, MZ1 is particularly efficacious in reducing MYCN in neuroblastoma [228]. Other reports indicate that BET degradation stabilizes MYC, which partially explains some disease‐dependent effects [237].

As seen in BET inhibition, the biological effects BRD4‐selective degradation approaches also diverge from effects seen with pan‐BET approaches. A clear example of this is through nuclear condensates where (+)‐JQ1 was unable to displace BRD4 from super enhancer regions [232]. BRD4‐selective degradation through ZXH‐3‐26 initially led to the loss of both MED1 and p‐TEFb (responsible for transcriptional elongation and the recruitment of RNA Pol II when bound to BRD4) from these regions, however, these factors returned over time despite sustained BRD4 degradation. This result suggests that although BRD4 may initially contribute to condensate formation, alternative compensatory mechanisms may arise over time. Other BRD4‐selective inhibitors have also shown efficacy in breast‐cancer cell lines [180, 238]. In the case of dBRD4, we initially investigated its effect on c‐Myc in a multiple myeloma model (MM1.S). However, BRD4‐selective degradation did not affect c‐Myc expression until micromolar levels despite a DC_50_ = 280 nM for BRD4. At these elevated concentrations, the selectivity of the parent ligand (iBRD4‐D1) erodes, leading to a pan‐D1 inhibition profile. In this case, degradation of BRD2 and BRD3 were not observed, supporting that pan‐D1 inhibition was the more likely cause of the observed c‐Myc depletion. While we did not initially observe an increase in c‐Myc levels as reported with BRD4‐D1 inhibition [160], BRD4‐selective degradation led to a slight increase of BRD2 and BRD3 levels. Notably, the upregulation of the other BET proteins was not observed with either MZ1 or ZXH‐3‐26. This may indicate a possible compensation mechanism within the BET family; however, more thorough examination of this system is needed. As BRD4‐selective approaches in inhibition and degradation are now emerging, further investigation into the mechanisms of BET proteins in multiple myeloma may be necessary to achieve clinical success. The use of selective PROTACs and inhibitors provide versatile tools to investigate the differences within the BET family in these models, which will inform disease indications and clinical efforts.

Despite concerns of BET‐related toxicities, the sub‐stoichiometric mode‐of‐action that PROTACs offer enables lower dosing regimens, combined with the proven efficacy of BET‐targeting approaches. These factors together have enabled two BET‐targeting PROTACs of interest that are currently in clinical trials: MT4561 (NCT06943521) and RNK‐05047 (NCT05487170, Figure 8) [239, 240]. MT4561 is currently in Phase I/II in order to evaluate safety, tolerability, and efficacy in patients with advanced solid tumors, including NUT carcinomas, breast cancer variants, non‐small cell lung cancer (among others). Previously, MT4561 was disclosed to be particularly effective in degrading the BRD4‐NUT fusion protein in addition to BRD3‐NUT cells, leading to c‐Myc suppression in vivo [241]. Likewise, RNK‐05047 is under evaluation for patients with diffuse large B‐cell lymphoma. With their unique mode‐of‐action compared to pan‐BET inhibition, it remains to be seen if these PROTACs will show the required safety and efficacy needed to bridge the gap between BET efficacy and BET‐related toxicities. While other PROTACs are currently further in clinical trials, those targeting the BET bromodomains are only now emerging onto the scene. Whether these approaches will limit dose‐limiting toxicities remains to be seen, though alternative delivery mechanisms that allow for tissue‐selective delivery allow for the growth and exploration of these fields.

## 9 PROTACs Developed for Non‐BET Bromodomain‐Containing Proteins

**FIGURE 10:**
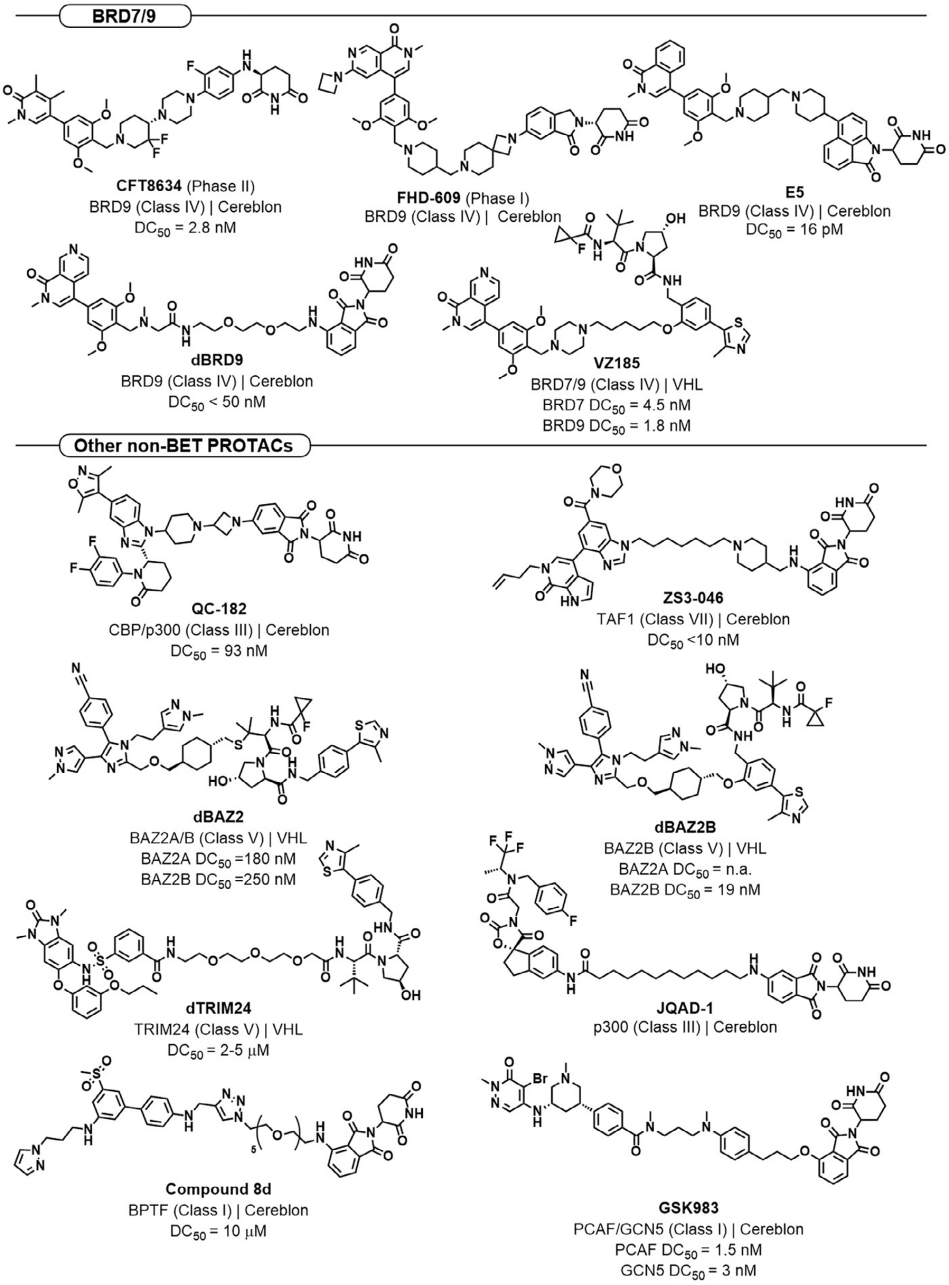
Structures of representative PROTACs targeting non‐BET proteins with their respective selectivity, potency, and clinical trial status, compound structures are arranged so that the target‐binding ligand is oriented on the left and the E3‐ligase biding ligand is oriented on the right of each structure. DC_50_ values are listed where available.

While BET‐targeting PROTACs have emerged as a popular therapeutic modality along with other important biomarkers (such as the estrogen and androgen receptors), PROTACs have also been developed for non‐BET bromodomain‐containing proteins [62]. Notably, several non‐bromodomain targeting PROTACs are currently in clinical trials, which have been excellently reviewed elsewhere; this review will focus primarily on bromodomain‐containing approaches [190, 192, 242]. The PROTACs developed include primarily those with ligandable domains discussed previously, and are thus mostly limited to Class I, Class III, and Class IV bromodomains (Figures 4 and 10). However, these heterobifunctional modalities have shown significant promise for disrupting bromodomain‐containing proteins within large chromatin remodeling complexes. PROTACs targeting the BAF and SWI/SNF remodeling complexes are shown by BRD7/9 dual‐targeting PROTACs have been described, where compound VZ185 was efficacious in other leukemia models [243]. BRD9‐selectivity has also been conferred from E3‐ligase recruiting ligands, shown by the development of dBRD9 (Figure 10) [244]. In the same seminal work, Remillard et al. demonstrated additional selectivity for BRD9 over BET proteins using a thalidomide‐based CRBN ligand over lenalidomide‐based constructs, highlighting the role of the ternary complex. Remarkably, degradation of BRD9 and its scaffolding functions had significantly improved efficacy compared to bromodomain inhibition alone. One example of this is from Duan et al. with compound E5, where BRD9 degradation was 1000‐fold more potent at reducing the viability of the diffuse large B‐cell lymphoma line, OCI‐LY10 [123]. In the case of MOLM‐13 cells, BRD9‐selective degradation was >3000‐fold more efficacious compared to BRD9 bromodomain inhibition with I‐BRD9 (EC_50_ = 0.37 and 1219 nM, respectively). As mentioned above, BRD9 inhibition alone did not induce a significant phenotypic response, but rather the BRD9 degradation was needed. The therapeutic significance of targeting BRD9 through protein degradation is shown by the clinical efforts made to advance PROTACs in these clinical settings [245, 246, 247]. Thus, PROTACs targeting these epigenetic regulators in large chromatin remodeling complexes can serve as potential therapeutics, where selective approaches aid in elucidating bromodomain‐containing proteins’ scaffolding roles in these complexes.

For other non‐BET bromodomain‐containing proteins, the development of bromodomain‐targeting PROTACs has also extended to CBP/p300 with the development of QC182 (Figure 10), which was more effective than bromodomain CBP/p300 inhibition in hepatocellular carcinoma [248]. Selectivity for p300 over CBP has also been achieved through small‐molecule‐induced degradation, where JQAD‐1 was used to determine the role of p300 in the enhancer landscape of neuroblastoma [84]. Recently, a selective peptide‐based PROTAC targeting p300 was also reported, where the selective degradation of p300 showed efficacy in both androgen‐receptor positive and negative cell lines and led to a substantial reduction in c‐Myc and increased levels of apoptosis [249]. However, several other non‐selective SMARCA2/4 PROTACs have been reported in patents and literature, which have been efficacious in a variety of disease states [250, 251, 252, 253, 254]. Potent targeted‐protein degradation modalities have also been reported for Class V and VII bromodomain‐containing proteins, BAZ2A/B and TAF1. Targeting BAZ2 proteins, Palaferri et al. recently developed the PROTAC dBAZ2, and while not isoform‐selective, degrades BAZ2A and BAZ2B with a DC_50_ = 180 and 250 nM, respectively [255]. This also led to the development of the isoform‐selective PROTAC, dBAZ2B, which optimized the exit vector from the VHL‐recruiting ligand. In PC3 cells, a prostate cancer cell line, dBAZ2B was isoform‐selective in its degradation of BAZ2B over BAZ2A while maintaining its efficacy (DC_50 _= 19 nM), likely as a result from productive ternary‐complex formation or selective ubiquitination. The Class VII bromodomain‐containing protein, TAF1, also represents a promising target for protein degradation, as it contains a set of tandem bromodomains and several other enzymatic domains (including two potential kinase domains and a HAT domain). As such, degradation of TAF1 with ZS3−046 resulted in activation of p53 in in vivo models of AML, whereas bromodomain inhibition alone was unable to achieve these effects [102, 256]. While current approaches to targeting MLL do not include small‐molecule bromodomain inhibition, PROTAC approaches targeting this complex have focused on the other domains of this therapeutically significant complex [257]. Given bromodomain‐containing proteins’ propensity to modulate gene transcription, alternative therapeutic modalities to bromodomain inhibition are being explored. These include both targeted protein degraders (in the form of PROTACs) or targeting other enzymatic domains of these proteins. Examples include the development of MLL methyltransferase inhibitors [258] and SMARCA2/4 ATPase inhibitors [61], where these approaches were efficacious against cancer [259]. These results support the potential for targeting these classes of bromodomain‐containing proteins and motivate development of bromodomain‐targeting ligands for these underexplored families.

For the Class VII bromodomain‐containing protein, TRIM24, the PROTAC dTRIM24 was also developed (Figure 10) [260]. This led to TRIM24's identification as a novel leukemic target. Importantly, ligands that target multiple domains of TRIM24 have recently been shown to be efficacious [76], where PROTACs also address the therapeutic benefit and function of multiple domains within these epigenetic regulators. The ability of PROTACs to address additional scaffolding function within these complexes remains one of their strengths in epigenetic therapies. This concept has also been applied to target a member of the NURF complex, BPTF. Here, several attempts to degrade BPTF have been applied, leading to the development of Compound 8 and Compound 8d [261, 262]. Indeed, Compound 8 is potent against CECR2 at nanomolar concentrations. Further, BPTF‐degradation through Compound 8d enhanced response to NK‐cell based immunotherapies. Additional examples of targeting Class I bromodomain‐containing proteins include the development of GSK983, which degrades both PCAF and GCN5 [263]. The degradation of PCAF/GCN5 was shown to be particularly effective at reducing IL‐6 production in macrophages and dendritic cells, implicating their role in immune response. The innovation of PROTACs targeting non‐BET bromodomain‐containing proteins has only recently begun, where the further development of protein degradation may be particularly efficacious given bromodomains’ role in chromatin remodeling complexes.

## 10 Summary and Outlook

From a phenotypic standpoint, the BET family has remained a significant point of interest in the field, where more selective inhibitors and PROTACs serve as useful approaches for deciphering the role of each BET protein in varied disease indications. The development of both inhibitors and PROTACs with enhanced selectivity within the BET family has allowed for the focused investigation into intra‐BET mechanisms and the roles of individual bromodomains, where inhibition and degradation of the BET family have previously shown distinct biological effects toward Myc [237, 264]. Inhibition of BET D1s appears to be broadly efficacious and tends to phenocopy pan‐BET inhibitors. Unfortunately, these approaches are continually held back by BET‐mediated toxicities akin to pan‐BET approaches. While targeting D2 domains within BET proteins has consistently led to more tolerated compounds, their effects on cellular proliferation are often reduced or are highly disease‐dependent. This is especially apparent in BRD4‐D2 inhibition [162]. Both BET D1‐ and D2‐targeting approaches are being investigated in the context of inflammation, as well, which also seems to be model‐dependent as far as efficacy is concerned [63, 139, 141, 265]. To further advise therapeutic development and to determine the extent of BET‐mediated toxicity, the next stage of understanding BET biology lies in understanding the role of individual domains and individual proteins, which remains unmet. BRD4‐selective approaches in combination to genetic knockdown have aided in elucidating some of these subtleties [144, 231], but more work remains in determining the differential effects in various disease indications. From a development point‐of‐view, several alternative modalities to small molecule inhibition have come to light, often utilizing PROTACs [190]. One interesting example is with antibody‐drug conjugates in breast cancer models for tissue‐specific targeting to mitigate BET‐driven toxicities [266]. With emerging breakthroughs in selective inhibition and degradation, as well as selective tissue targeting, small molecules and PROTACs are now selective enough to serve as useful model compounds for deciphering mechanistic effects of BET proteins. Through these combined approaches, it is likely that clinical breakthroughs can be achieved through the thorough understanding of BET biology in both cancer and inflammation, where the promise of BET‐targeting therapies merits further investigation.

For non‐BET bromodomain‐containing proteins, the investigations into these emerging epigenetic therapies continue to further the understanding of bromodomains as functional motifs in the role of chromatin remodeling, highlighted by emerging clinical data. Indeed, the role of non‐BET bromodomain‐containing proteins may be an alternative approach to circumvent the clinical toxicities seen by pan‐BET inhibition in some disease indications; however, bromodomain inhibition alone may be insufficient for these proteins in order to achieve the desired therapeutic effects. For example, the role of BPTF and NURF in cancer continues to be investigated through both chemical inhibition and PROTAC modalities, potentially offering alternative therapeutic targets to BET proteins [11, 119, 262]. Other bromodomain‐containing remodeling complexes, such as CECR2 present in CERF, continue to be investigated for a variety of developmental, congenital, and DNA repair mechanisms [267]. The continued investigation into these bromodomain‐containing proteins also reveal distinct molecular mechanisms for scaffolding chromatin remodeling complexes, where bromodomain inhibition may not significantly reduce downstream gene transcription [58, 123]. Further, both bromodomain inhibitors and PROTACs continue to be developed, where a large subset of human bromodomains have been investigated due to their biological significance. For bromodomains that remain more elusive for small‐molecule approaches, genetic knockdown approaches or peptide‐based ligands may be useful in elucidating the role of these bromodomains in epigenetic mechanisms in addition to the PROTAC strategies that continue to emerge. This includes structurally similar bromodomains, such as efforts in developing p300 selectivity through targeted degradation [249]. As more cell‐active chemical probes are developed for these bromodomain‐containing proteins, we can further understand the biological mechanisms that underpin epigenetic regulation and the histone code. This includes the subtle biological differences between structurally similar bromodomain‐containing proteins, such as CBP/p300, BRD7/9, and PCAF/GCN5 [79, 98, 122, 123, 263]. More information regarding the cooperativity between these non‐BET bromodomains and allosteric functions can also be determined through the use of PROTACs, or as ligands continue to develop for other epigenetic domains (i.e., PHD, HAT). Indeed, the role of these non‐BET bromodomain‐containing proteins still represents a particular gap in the field, though more well‐established proteins such as CBP/p300 continue to show the biological importance of the non‐BET bromodomain‐containing proteins.

## Author Contributions

**Cole R. Scholtz:** conceptualization, writing – original draft, writing – review and editing, data curation, formal analysis. **William C. K. Pomerantz:** conceptualization, writing – original draft, writing – review and editing, data curation, formal analysis, funding acquisition.

## Funding

This study was supported by National Institute of General Medical Sciences (Grants R35 GM140837, T32GM008347).

## Conflicts of Interest

William C. K. Pomerantz is a co‐founder of BromoThera.

## Data Availability

The data that support the findings of this study are openly available in clinical trials at http://clinicaltrials.gov/.
